# Historical anthropogenic disturbances explain long‐term moorland vegetation dynamics

**DOI:** 10.1002/ece3.9876

**Published:** 2023-03-08

**Authors:** Francis M. Rowney, Ralph M. Fyfe, Leonard Baker, Henry French, Martha B. Koot, Havananda Ombashi, Rhys G. O. Timms

**Affiliations:** ^1^ School of Geography, Earth and Environmental Sciences University of Plymouth Plymouth UK; ^2^ Department of History University of Exeter Exeter UK; ^3^ School of Culture and Society Aarhus University Aarhus Denmark; ^4^ Quaternary Scientific, (Quest), School of Archaeology, Geography and Environmental Science University of Reading Reading UK

**Keywords:** coprophilous fungal spores, interdisciplinary, moorlands, Paleoecology, peatlands, pollen

## Abstract

Upland moorlands are important landscapes, but many are considered degraded as a result of human activities. Consequently, their protection and restoration are of substantial concern. In Europe, restoration activities are often aimed at reversing the effects of 19th and 20th century “agricultural improvements,” which often involved major drainage schemes. However, the ecological effects and long‐term ecological context of “agricultural improvement” are not yet fully understood. To develop this understanding, we analyze paleoecological data (pollen, coprophilous fungal spores, microcharcoal) from five upland peatland sites using a range of analytical approaches: cluster analysis, principal component analysis, rate‐of‐change analysis, and regression analyses incorporating documentary historical data. The sites are located on Exmoor (South West England, UK), a landscape that typifies historic upland degradation. We demonstrate that in this landscape, 19th century drainage is associated with declines in *Sphagnum* and non‐arboreal taxon richness; over longer timescales burning is associated with enhanced graminoid monocot abundance and grazing with lower taxon richness. We also show that rate‐of‐change in moorland vegetation communities during the 19th century is not distinctive in a long‐term context: change has been a constant in this landscape, rather than an exception during the 19th century. Our findings indicate that the aims of “restoration” interventions intended to increase *Sphagnum* abundances, increase taxon richness and reduce graminoid dominance are consistent with the long‐term dynamics of peatland systems, such as those on Exmoor. “Restoration” deemed successful in these terms may or may not resemble pre‐drainage conditions, which were themselves a function of millennia of successive moorland management regimes.

## Graphical abstract

The ecological impacts of 19th century “agricultural improvements” are not yet fully understood, but this is important for moorland restoration. Paleoecological and historical analyses demonstrate specific 19th century ecological changes in a landscape that typifies historic upland degradation. These changes are placed within a context of long‐term anthropogenic disturbance.

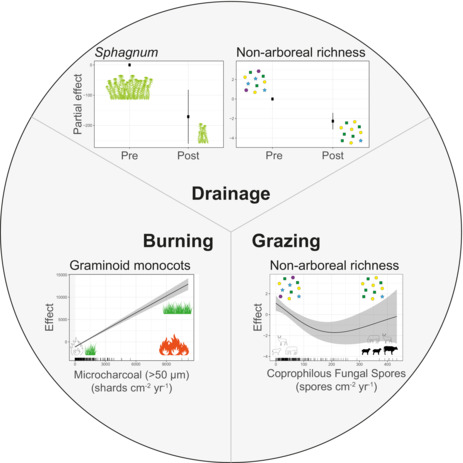

## 1 INTRODUCTION

Moorland landscapes are important landscapes for their roles in supporting climate change mitigation, water resource management, specialist biota, and biocultural heritage, particularly in areas with extensive peatlands (Grand‐Clement et al., 2013; Littlewood et al., 2010; Minayeva et al., 2017; Ritson et al., 2016; Rotherham, 2015; Zak & McInnes, 2022). Consequently, their protection and restoration are of substantial concern, as many are considered to be degraded as a result of human activities such as drainage, burning, and peat cutting (Grand‐Clement et al., 2013; Leifeld & Menichetti, 2018; Tanneberger et al., 2021). Macro‐ecological studies have demonstrated the influence of different management and environmental conditions on the vegetation of peatlands (Yeo & Blackstock, 2002), although the role of particular disturbance agents (e.g., fire) remains controversial (Davies et al., 2016). Long‐term data spanning historical timescales offer important insights into disturbance regimes, but are limited to re‐surveys that do not extend beyond ~60 years into the past (Alday et al., 2022; Britton, Hester, et al., 2017; Britton, Hewison, et al., 2017). The initial impacts of important historical disturbance regimes, such as enclosure and drainage, are not captured in such datasets, risking inappropriate benchmarking in measurements of ecosystem recovery or condition. Long‐term data from paleoecology offer approaches with potential to resolve this problem (Seddon et al., 2014). In this paper, we combine such paleoecological data with historical information to address the extent to which anthropogenic disturbance regimes impacted peatland character, drawing on data from Exmoor, South West England.

The 19th century was a period of substantial change in terms of society, economy, and land governance, with Parliamentary enclosure legislation between 1760 and 1840 CE leading to major landscape change. Enclosure on Exmoor represents one of the most ambitious projects of the time: *~*60 km^2^ was purchased by John Knight in 1818 CE and subjected to “agricultural improvement” schemes (Orwin & Sellick, 1970). Management practices, particularly related to drainage and irrigation for “agricultural improvement,” are well‐documented (Hegarty & Wilson‐North, 2014; Orwin & Sellick, 1970; Riley, 2019; Riley & Wilson‐North, 2001), and some associated changes in vegetation and microbial ecology have been noted at individual sites (Chambers et al., 1999; Francis & Slater, 1990; Fyfe, Brown, & Rippon, 2003; Rowney et al., 2022). Restoration work aimed at reversing the effects of drainage on Exmoor has been ongoing since 2010 (Gatis et al., 2016; Grand‐Clement et al., 2015). Vegetation is monitored in restoration areas but the ecological impacts and long‐term ecological context of 19th century “agricultural improvements” are not fully understood. To develop this understanding, we address the following questions, using paleoecological analyses spanning the period of time before, during, and after “improvement,” and incorporating documentary historical data:

1. To what extent have grazing, burning and drainage been associated with ecological change on Exmoor peatlands during the last *~*600 years?
2. Was the 19th century a notable period of ecological change on Exmoor, in terms of character and/or rate‐of‐change?
3. Are the aims of restoration activities on Exmoor consistent with long‐term vegetation dynamics?

We focus specifically on *Sphagnum* abundance, graminoid monocot abundance, and taxon richness. Each of these variables is linked to specific ecosystem services or stakeholder interests: *Sphagnum* is a keystone taxon that plays a central role in carbon sequestration and water resource management (Bacon et al., 2017; Grand‐Clement et al., 2013; Ritson et al., 2016); the mono‐dominance of graminoids (e.g., *Molinia caerulea*, *Eriphorum vaginatum*) in upland habitats is of notable concern to land managers (Glaves, 2016; Lunt et al., 2021; Marrs et al., 2004; Meade, 2016); biodiversity (taxon richness) has broad societal value and provides a whole‐community perspective (Littlewood et al., 2010; Minayeva et al., 2017).

## 2 MATERIALS AND METHODS

Key methodological details are provided here, and further details are given in the Appendix S1.

**FIGURE 1:**
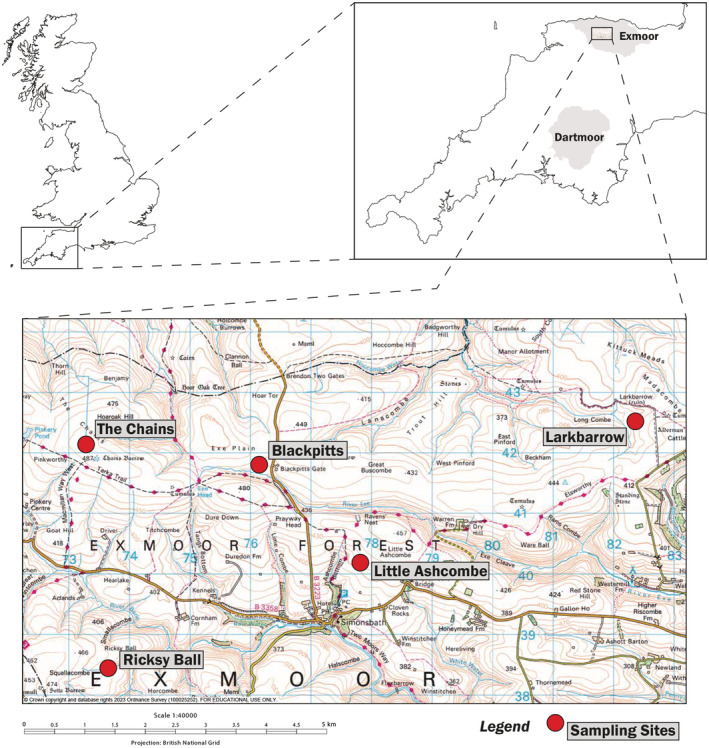
Map of paleoecological sampling sites.

Samples for paleoecological analyses were collected from five upland peatland sites within the Royal Forest area of Exmoor: Blackpitts, Larkbarrow, Little Ashcombe, Ricksy Ball, and The Chains (Figure 1).

### 2.1 Chronology

The peat sequences were dated using a suite of methods: AMS (accelerator mass spectrometry) radiocarbon analysis, fallout radionuclide (^210^Pb and ^137^Cs) analysis, and tephrochronology. Age‐depth models were constructed from robust dates using the R (R Core Team, 2022) packages “rplum” (Blaauw et al., 2021) (Blackpitts, Larkbarrow, Little Ashcombe) and “rbacon” (Blaauw et al., 2022) (The Chains) (see Rowney et al., 2022, for Ricksy Ball). Radiocarbon dates were calibrated using the IntCal20 calibration curve (Reimer et al., 2020).

### 2.2 Paleoecology

Pollen, coprophilous fungal spore, and microcharcoal samples were prepared and recorded simultaneously (i.e., the same samples, preparation, and slides were used), using standard pollen extraction methods (Moore et al., 1991). Known quantities of an exotic marker (*Lycopodium* spores) were added to allow the calculation of concentrations and influx rates (Stockmarr, 1971). Typically, 300 land pollen grains were identified per sub‐sample: a sufficient number for characterizing assemblages (Djamali & Cilleros, 2020). Three fungal spore types demonstrably associated with dung (*Sporormiella*‐, *Podospora*‐ and *Sordaria*‐types) (Baker et al., 2013; Perrotti & van Asperen, 2018) were recorded. Their abundances are reliable correlates of moorland stocking densities, particularly when multiple types are considered simultaneously (Davies et al., 2022). Microcharcoal abundances were estimated by counting charcoal particles (shards) >50 μm (length).

### 2.3 Archival data

**FIGURE 2:**
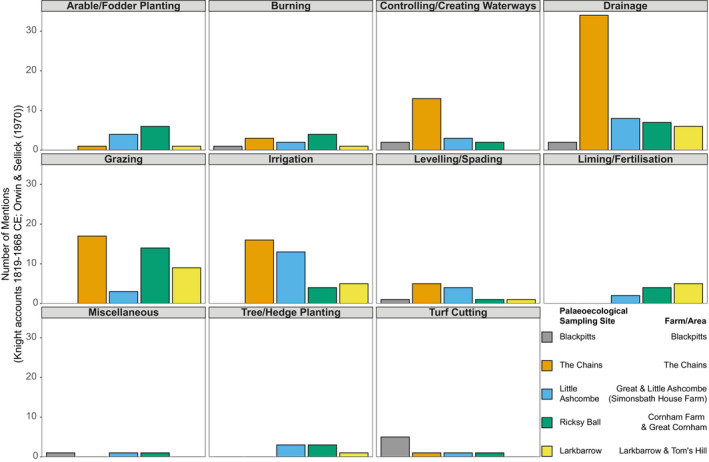
Summary of documented activities for each area relevant to paleoecological sampling sites (see Table S1).

Documentary evidence for interventions related to 19th century “agricultural improvement” has been drawn from the “Knight family archive” (contemporary financial accounts and correspondence) (Wilson‐North, 2017) and other sources (see Appendix). These are constrained to specific times (years, often months) and areas (e.g., tenanted farms) and include a range of activities with potential for inclusion in subsequent quantitative analyses (see Figure 2 and Table S1). Paleoecological sampling sites can be confidently related to farms/areas mentioned (see Figure 2), though the lack of accompanying contemporary mapping means that the precise spatial extents of these farms/areas are difficult to determine. Drainage is consistently recorded at each site (see Figure 2), and as there is no evidence for extensive drainage on Exmoor prior to the 19th century (MacDermot, 1973; Orwin & Sellick, 1970), this provides confident dates for the earliest moorland drainage in each area. Grazing and burning have substantially longer histories (Fyfe et al., 2018; Riley & Wilson‐North, 2001), and archival data are insufficient to represent these activities for the study period (*~*600 years). Other activities that are unlikely to have taken place prior to the 19th century (e.g., liming) are less consistently recorded and are unsuitable for subsequent analyses. These data demonstrate the breadth of activities undertaken during this period (illustrating that disturbance was not limited to drainage), but they cannot be considered fully comprehensive records, as the accounts and correspondence are dependent on different motivations, incentives, and tenancies (see Appendix S1).

### 2.4 Cluster analysis

Cluster analysis was used with rate‐of‐change analysis (see below) to classify groups of samples with similar paleoecological assemblages and to identify clusters associated with periods of rapid ecological change. Analyses were performed using the “vegan” (Oksanen et al., 2022) and the R function “hclust” (R Core Team, 2022).

### 2.5 Ordination

Unconstrained ordination was used to assist in characterizing clusters. Detrended correspondence analyses (DCA) were performed to determine environmental gradient lengths, and all gradient lengths were <2.5 standard deviations. On this basis, a linear method (PCA) was selected (following Legendre and Birks (2012)). To aid interpretability, data were filtered to include only common taxa (those occurring in ≥25% of samples) prior to analysis (the inclusion of more taxa in sensitivity testing made little difference), and abundances (influx rates) were square root transformed (to facilitate analyses based on the direction and relative magnitude of changes). Analyses were performed using the R package “vegan” (Oksanen et al., 2022).

### 2.6 Rate‐of‐change analysis

Rate‐of‐change analysis was used to explore the pace of ecological change through time, and in particular, whether 19th century rate‐of‐change is notable in a long‐term context (*~*600 years). Analyses were performed using the R package “R‐Ratepol” (Mottl et al., 2021).

### 2.7 Regression modeling

Regression modeling was used to assess associations between three response variables representing different aspects of moorland vegetation (*Sphagnum* spore influx, monocot pollen influx, non‐arboreal taxon richness) and several explanatory variables representing potential controls (see Table 1). Generalized additive models (GAMs) were used for these analyses, using the R package “mgcv” (Wood, 2022). GAMs are a flexible extension of generalized linear models (GLMs), which do not assume linearity in the relationships between the mean of the response and the explanatory variables (Birks, 2012; Wood, 2017). The effects of the explanatory variables on the mean response are defined by “unknown” smooth functions that are estimated from the data, rather than being assumed (e.g., linear) a priori. As such, GAMs are considered “data‐driven” models (Birks, 2012). The construction of GAMs for each response variable was broadly similar: All explanatory variables in Table 1 were included, as well as random effects for sampling sites.

**TABLE 1:** Response and explanatory variables in generalized additive models (GAMs) (summary statistics: Table S2).

Variable		Description	
Response	*Sphagnum* abundance	*Sphagnum* spore influx (spores cm^−2^ yr^−1^)	Continuous
	Graminoid monocot abundance	Poaceae and Cyperaceae pollen influx (grains cm^−2^ yr^−1^)	Continuous
	Taxon richness (diversity)	Non‐arboreal pollen taxon richness (the number of non‐arboreal pollen types identified per sample)	Continuous
Explanatory	Summer precipitation	Reconstructed based on stable oxygen isotopes in tree rings (Loader et al., 2020), 1201–2000 CE.	Continuous
	Grazing (coprophilous fungal spores)	*Sordaria*‐type, *Sporormiella*‐type, and *Podospora*‐type influx (spores cm^−2^ yr^−1^)	Continuous
	Burning (microcharcoal)	Microcharcoal (>50 μm) influx (shards cm^−2^ yr^−1^)	Continuous
	Drainage	Binary variable (“pre‐drainage” or “post‐drainage”) based on earliest record of drainage per site.	Categorical
	Site type	Binary variable (“ombrotrophic” or “soligeneous”), accounting for differences in pollen catchment area.	Categorical

## 3 RESULTS

### 3.1 Chronology

Sequences were dated using a combination of methods (see Table 2). Age‐depth models were constructed for each site using this combination of datasets (details in Figures S3–S6). Chronological details for Ricksy Ball have been published (Rowney et al., 2022).

**TABLE 2:** Summary of dating methods.

Site	Radiocarbon dates (Table S3)	Fallout radionuclide (^210^Pb, ^137^Cs) (Table S4)	Tephras (with ages) (Table S5; Figures S1 and S2)
Blackpitts	13	Yes	3
Larkbarrow	5	Yes	0
Little Ashcombe	6	Yes	2
Ricksy Ball^a^	8	Yes	2
The Chains	6	No	0

^a^ Rowney et al. (2022).

### 3.2 Paleoecological assemblages

The pollen data (Figures S7–S10; Rowney et al., 2022) show that the five sites have been open moorland for at least the last ~600 years, though each site has differed in the precise characteristics of local moorland vegetation and disturbance regimes through time. The relative abundances of major vegetation types vary in space and time, but the major taxa present are broadly consistent: grasses (Poaceae); sedges (Cyperaceae); heathers (*Calluna vulgaris*, Ericaceae undiff.); herbaceous taxa associated with open habitats (*Plantago lanceolata*, *Potentilla*‐type, *Rumex acetosa/acetosella*); trees and shrubs, most likely in the surrounding landscape (*Betula*, *Corylus avellana*‐type, *Quercus*).

**FIGURE 3:**
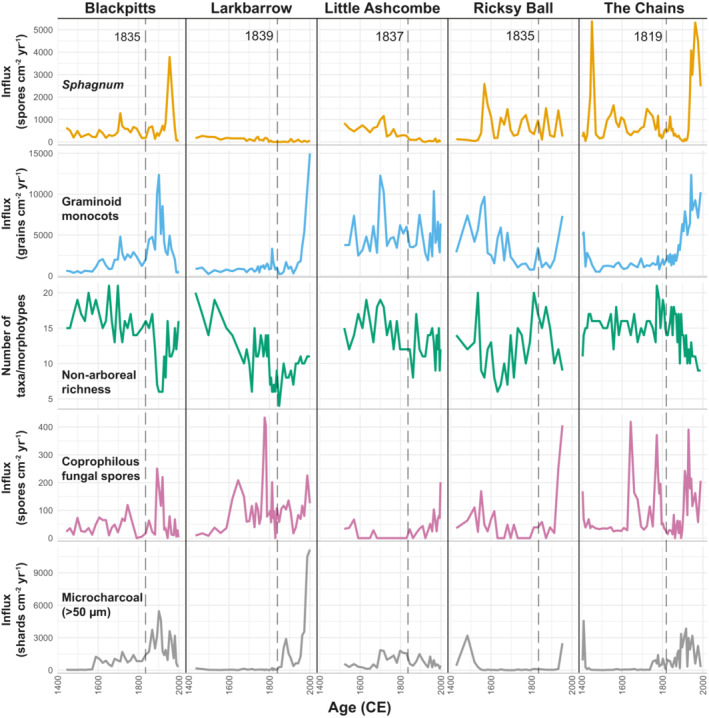
Summaries of key variables used in regression modeling (dashed lines represent earliest records of drainage per site).

Figure 3 highlights the paleoecological variables used in regression modeling: *Sphagnum* and graminoid monocot (Poaceae and Cyperaceae) influx rates, non‐arboreal taxon richness, coprophilous fungal spore influx, and microcharcoal (>50 μm) influx. In general, temporal trends in these variables are site‐specific, rather than following a common pattern, though some similarities are clear. The influx rates of *Sphagnum* and coprophilous fungal spores are particularly site‐specific, likely reflecting different hydrological settings and varying land use through time at each site. Graminoid monocot pollen and microcharcoal influx show similarities between sites and are both generally higher (or increase) during the nineteenth and twentieth centuries. Non‐arboreal taxon richness is variable in space and time, but follows a general pattern of decline (though there is substantial complexity within this), particularly during the nineteenth and/or twentieth centuries.

### 3.3 Cluster, ordination, and rate‐of‐change analyses

**FIGURE 4:**
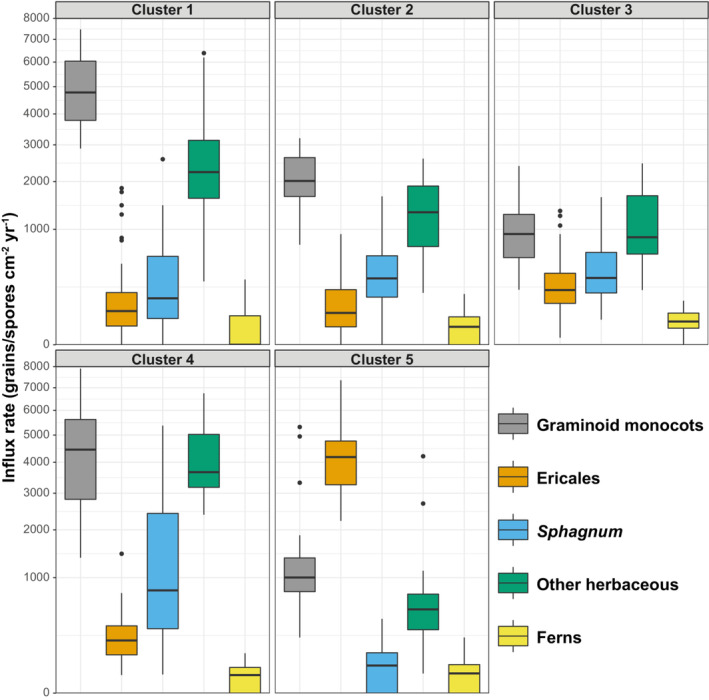
Summaries of clusters based on vegetation groups (see also Figure S11).

**FIGURE 5:**
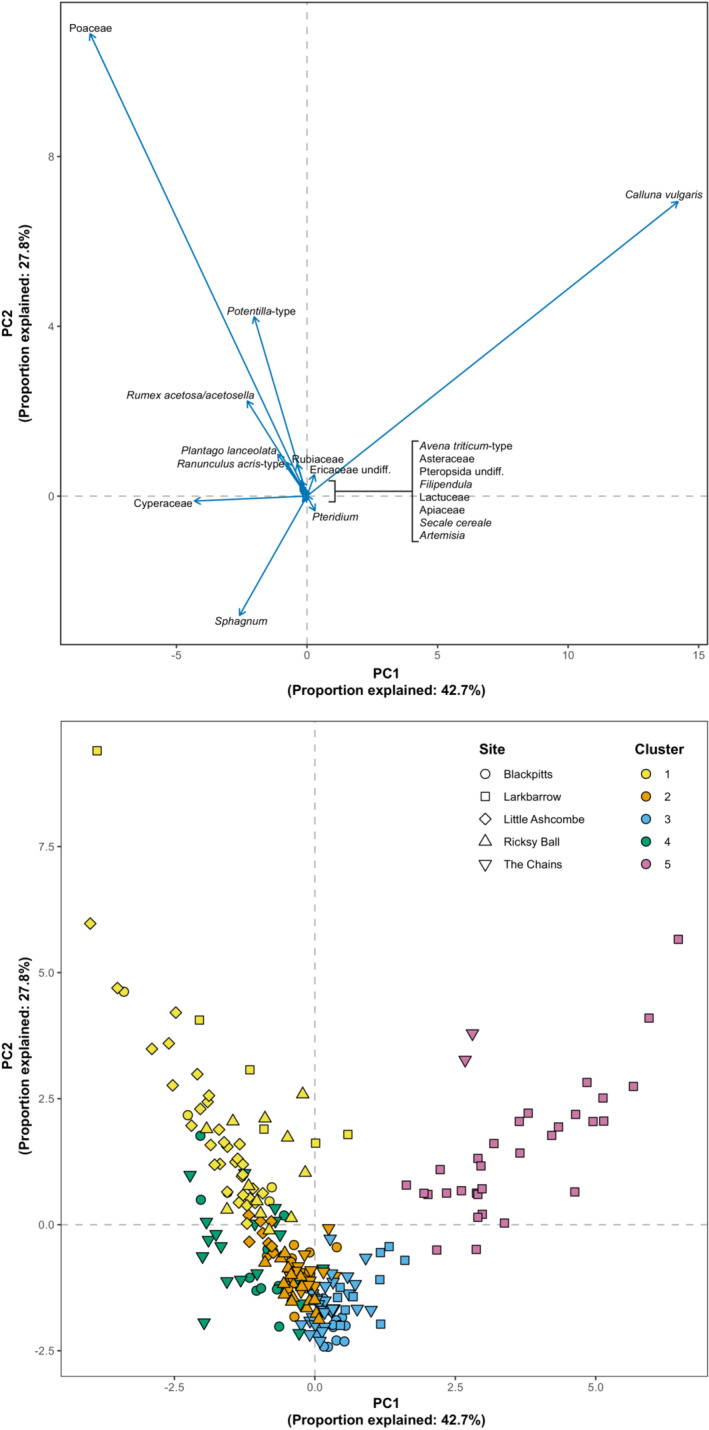
Principal component analysis (PCA) of non‐arboreal components of pollen assemblages (based on influx values).

Non‐arboreal components of pollen assemblages were divided into five clusters (corresponding to the number of sites, to allow for the potential that spatial clustering may be more important than temporal), the characteristics of which are summarized in Figures 4 and 5 (see also Figure S11). These appear to be broadly defined by the abundances of Poaceae and *Calluna vulgaris*. Clusters 1 and 4 have the highest proportions of graminoid monocots, particularly Poaceae (grass), alongside herbaceous taxa including *Plantago lanceolata, Potentilla*‐type, and *Rumex acetosa/acetosella*, while Clusters 2 and 3 show more even mixtures of monocots and other herbaceous taxa. Cluster 5 represents samples with high abundances of ericales (*Calluna vulagris*, Ericaceae undiff.), largely drawn from Larkbarrow.

**FIGURE 6:**
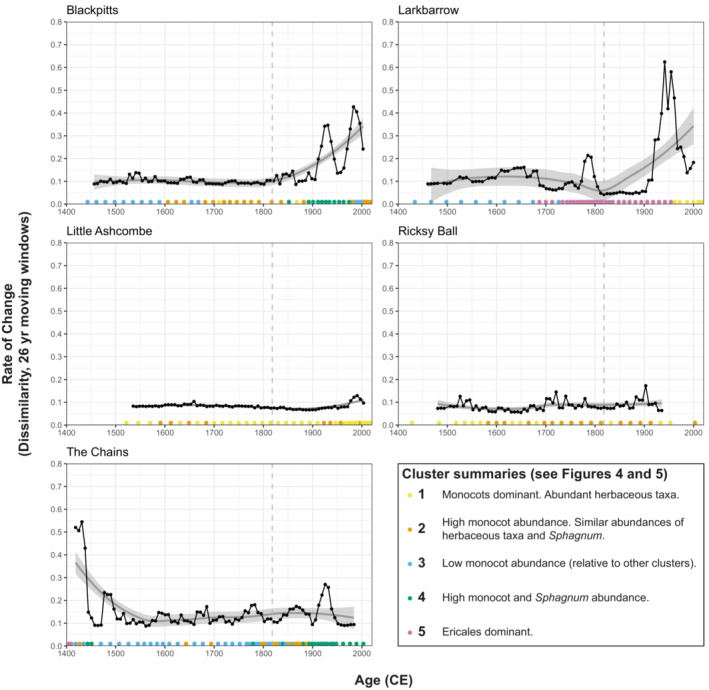
Rate‐of‐change plots for each pollen site, based on 26‐year moving windows. Rug plots of colored points represent pollen samples and their respective clusters (see Figures 4, 5, and S11). (The maximum time represented by a single pollen sample (according to the age‐depth models) is 25.6 years: moving windows (“bin_size”) of 26 years were used. “Number_of_shifts” was set to four, resulting in 6‐year estimates of rate‐of‐change. Settings refer to “R‐Ratepol” (Mottl et al., 2021)).

In general, temporal trends in rate‐of‐change are site‐specific, rather than following a common pattern (Figure 6). Some sites (Blackpitts, Larkbarrow) show distinctive increases in rate‐of‐change during the 20th century, while others show broadly consistent (Little Ashcombe, Ricksy Ball) or frequently fluctuating (The Chains) rate‐of‐change through time. There is no evidence for increased rates of change during the 19th century (relative to preceding or subsequent periods). As with rates of change, the patterns of clusters through time are also broadly site‐specific (Figure 6), rather than following a general pattern. However, the distinctive increases in rate‐of‐change during the 20th century at some sites (Blackpitts, Larkbarrow, and, to a lesser extent, The Chains) are all associated with shifts toward greater graminoid monocot abundances.

### 3.4 Regression analyses

**FIGURE 7:**
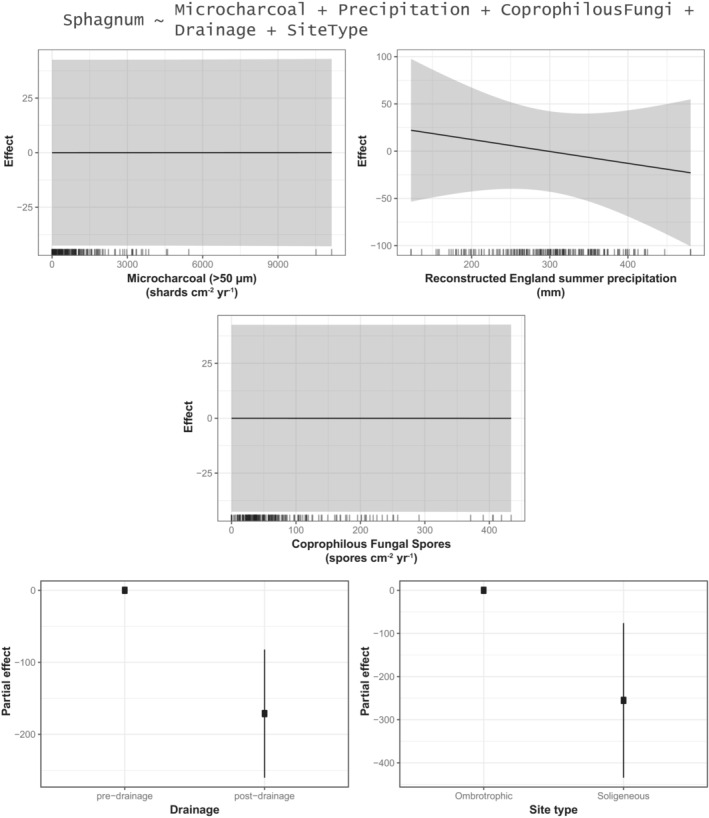
Graphical summary of a generalized additive model (GAM) (see Table S6) showing the estimated effects of microcharcoal (>50 μm) influx, summer precipitation, coprophilous fungal spore influx, drainage, and site type on Sphagnum spore influx (spores cm^−2^ yr^−1^) (see also Table 1; Figure 3).

**FIGURE 8:**
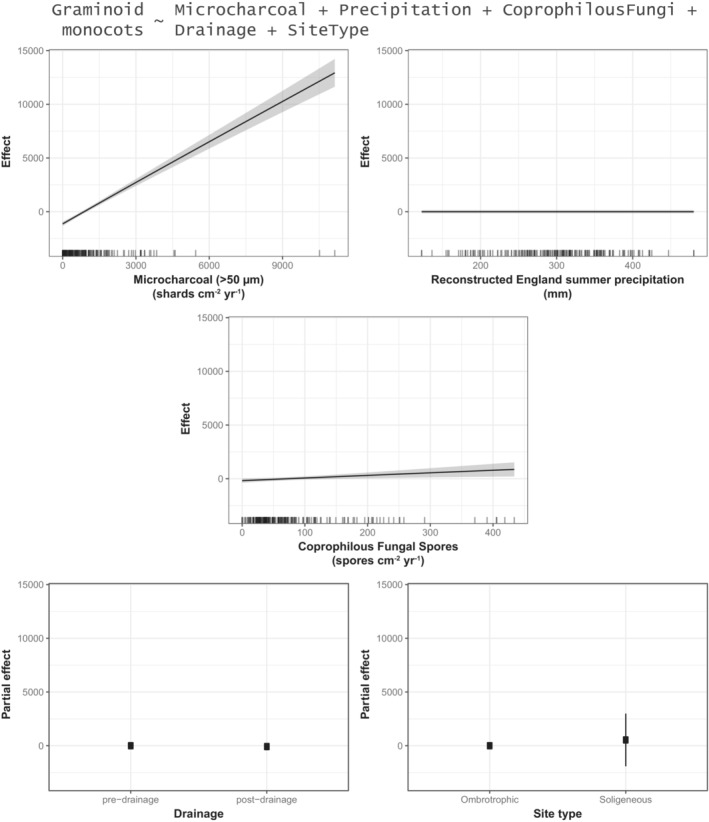
Graphical summary of a generalized additive model (GAM) (see Table S7) showing the estimated effects of microcharcoal (>50 μm) influx, summer precipitation, coprophilous fungal spore influx, drainage, and site type on graminoid monocot pollen influx (grains cm^−2^ yr^−1^) (see also Table 1; Figure 3).

**FIGURE 9:**
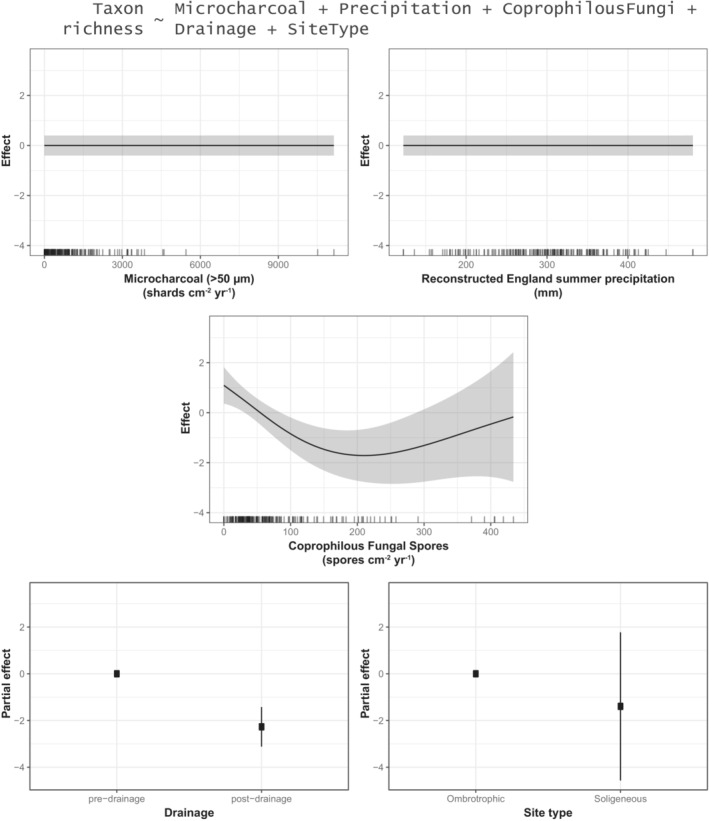
Graphical summary of a generalized additive model (GAM) (see Table S8) showing the estimated effects of microcharcoal (>50 μm) influx, summer precipitation, coprophilous fungal spore influx, drainage, and site type on non‐arboreal taxon richness (see also Table 1; Figure 3).

We demonstrate that relationships between moorland management and vegetation are not straightforward. Proxies of three different aspects of moorland vegetation (*Sphagnum* abundance, graminoid monocot abundance, taxon richness) (see Table 1) each have different relationships with proxies of three aspects of moorland management (burning, grazing, drainage) (see Figures 7, 8, 9, Tables 1 and S6–S8).

Nineteenth century drainage is significantly (negatively) associated with both *Sphagnum* spore influx (Figure 7) and non‐arboreal taxon richness (Figure 9), but it is not associated with graminoid monocot pollen influx (Figure 8). Relative to “pre‐drainage” pollen samples, “post‐drainage” samples are associated with −171.24 *Sphagnum* spores cm^−2^ yr^−1^ (±90.86, 2σ) (Figure 7) and −2.27 non‐arboreal taxa (±0.86, 2σ) (Figure 9). *Sphagnum* is also significantly associated with site type: relative to ombrotrophic sites (Blackpitts, The Chains), soligeneous sites (Larkbarrow, Little Ashcombe, Ricksy Ball) are associated with −255.22 *Sphagnum* spores cm^−2^ yr^−1^ (±182.88, 2σ) (Figure 7). In these models, “drainage” is represented by a binary variable, dividing samples into ~400 years before drainage and ~200 years following the first records of drainage for each site. Therefore, it is possible that these relationships may reflect longer term, centennial‐scale trends in *Sphagnum* abundance and taxon richness, rather than a direct effect of 19th century drainage. This is difficult to account for directly, as the linearly temporal nature of the “drainage” variable means that adjusting for time would potentially obscure any “real” effects of drainage on the outcome variables. In sensitivity testing, data were restricted to a shorter time period (1750–1900, *n* = 72) (Table S9), removing centennial‐scale trends preceding the 19th century, and any potential impacts of 20th century drainage. Statistically significant relationships with drainage remained for both *Sphagnum* and taxon richness, though each was slightly reduced. This suggests that 19th century drainage was associated with further declines in these aspects of moorland vegetation, in addition to longer term declines. However, site hydrology (site type) is likely a more significant influence on *Sphagnum* abundance.

Microcharcoal (>50 μm) influx is significantly (positively) associated with monocot pollen influx (Figure 8), but it is not significantly associated with either *Sphagnum* (Figure 7) or taxon richness (Figure 9). The maximum predicted effect on monocot pollen influx is +12927.52 pollen grains cm^−2^ yr^−1^ (±1323.95, 2σ) with 11138.55 microcharcoal (>50 μm) shards cm^−2^ yr^−1^, and the minimum is −1135.96 pollen grains cm^−2^ yr^−1^ (±191.16, 2σ) with 0 microcharcoal (>50 μm) shards cm^−2^ yr^−1^. This is a linear relationship (Figure 8), equating to an effect size of +1.26 monocot pollen grains cm^−2^ yr^−1^ per 1 microcharcoal (>50 μm) shard cm^−2^ yr^−1^. This suggests that moorland burning has promoted grass (Poaceae) and/or sedge (Cyperaceae) abundance on Exmoor.

Coprophilous fungal spore influx is significantly (non‐linearly) associated with non‐arboreal taxon richness (Figure 9), but it is not associated with either *Sphagnum* (Figure 7) or graminoid monocots (Figure 8). This relationship is negative from 0 to 208.83 spores cm^−2^ yr^−1^ (representing 92% of the dataset). Within this range, 208.83 spores cm^−2^ yr^−1^ is associated with −1.71 non‐arboreal taxa (±1.07, 2σ), and 0 spores cm^−2^ yr^−1^ is associated with +1.09 non‐arboreal taxa (±0.74, 2σ). At values >208.83 spores cm^−2^ yr^−1^ (maximum: 433.88 spores cm^−2^ yr^−1^), the relationship with taxon richness is uncertain. (Sensitivity testing, in which individual fungal spore types (*Sordaria*‐type, *Podospora*‐type, and *Sporormiella*‐type) were used instead of the total (Table S10), indicates that this relationship is primarily derived from the *Sordaria*‐type spores.) This suggests that increases in grazing pressure have been associated with decreases in non‐arboreal diversity on Exmoor, though little additional impact is associated with increases in coprophilous fungi beyond ~200 spores cm^−2^ yr^−1^. There is also a “significant” (*p* < .01) (positive) association with monocots, but the gradient of this relationship is weak to the extent that it can be considered negligible (Figure 8).

## 4 DISCUSSION

We present a detailed, interdisciplinary study of ecological change on a series of upland peatlands over the last ~600 years, incorporating historical data into paleoenvironmental analyses. We demonstrate the long‐term dynamism of upland peatlands, and that human interventions (drainage, burning, pastoral farming) had differential impacts on moorland vegetation through time. This provides vital long‐term data for ongoing management aimed at the “restoration” of peatland landscapes.

### 4.1 Was the 19th century a notable period of ecological change?

We integrate paleoecological and historical data to demonstrate that drainage is associated with distinct ecological changes in the peatlands studied here. Specifically, regression analyses show that post‐drainage landscapes are associated with lower *Sphagnum* abundances and pollen assemblages with fewer taxa (suggesting vegetation communities with fewer taxa) (Figures 7 and 9). *Sphagnum* is a non‐vascular plant, and so reduced abundance as a direct result of drying (drainage) is logical, and this may be also exacerbated by a number of cascading effects. Drainage ditches have long‐term effects on peatland hydrological processes, providing channels for water to flow more freely and making water tables less stable, as demonstrated by the effects of blocking them (Grand‐Clement et al., 2013; Wilson et al., 2010). Lower water tables lead to reduced resilience of peat to decomposition and burning (Granath et al., 2016; Harris et al., 2020). This can result in peat losses which in turn may lead to reduced water retention capacity, further exacerbating *Sphagnum* declines and hampering re‐growth. Lower water tables in upland peat systems are associated with greater abundances of *Molinia caerulea* (Purple Moor‐grass) (Gatis et al., 2016), which outcompetes other heathland plants (Lunt et al., 2021). It is challenging to identify the precise mechanisms leading to reduced diversity in post‐drainage settings, but enhanced *M. caerulea* abundance is likely to be a contributor, given the prevalence of Poaceae pollen in post‐drainage samples (and present abundance of this species). Other factors (e.g., grazing pressure) are also likely to be important. The number of non‐arboreal pollen taxa (taxon richness) is a reliable proxy of (relative) changes in vegetation diversity, though we acknowledge that there are limitations with this, particularly associated with taxonomic resolution (Odgaard, 1999). We did not find any association between *Sphagnum*, monocot graminoids, or diversity and a precipitation proxy, suggesting that anthropogenic disturbances may be more important in determining vegetation characteristics than precipitation in degraded peatlands. However, it should be noted that this is based on a regional proxy record (Loader et al., 2020), rather than a local precipitation reconstruction.

Regression analyses show that microcharcoal (>50 μm) is positively associated with monocot (graminoids: Poaceae and Cyperaceae) abundances (Figure 8), indicating that burning is likely to be another important control on Exmoor peatland vegetation during the last ~600 years. However, shifts toward grass‐dominated vegetation do not appear to be a 19th century phenomenon. Vegetation communities at most sites have developed consistently high grass abundances either before (Blackpitts, Little Ashcombe, Ricksy Ball) or after (Larkbarow, The Chains) the 19th century, rather than during (Figures 3 and 6). Positive relationships between burning and grass abundances have previously been demonstrated in Exmoor paleoecological records, although burning can also be associated with heather (*Calluna vulgaris*), and both relationships are variable in time and space (Fyfe et al., 2018). *Molinia caerulea* (Purple Moor‐grass) is of particular interest to land managers (Meade, 2016), and burning has been implicated in contributing to its present dominance in many moorland areas (Chambers et al., 1999), though this relationship is not straightforward (Glaves, 2016). *M. caerulea* was present on Exmoor prior to the 19th century (Chambers et al., 1999), but its dominance likely developed during the twentieth century in response to changes in burning, grazing and/or atmospheric pollution (Chambers et al., 1999, 2007, 2013; Chambers & McCarroll, 2016). On this basis, pre‐20th century monocot‐dominated vegetation communities in this study were likely characterized by other grasses. However, wild grasses cannot be readily discriminated beyond family‐level in pollen studies using standard microscopy (Mander et al., 2013), and this remains tentative.

The 19th century is demonstrably associated with specific ecological changes on Exmoor (see above), but is not unique in this regard. Exmoor has a long history of human habitation, disturbance, and management (Fyfe, 2012; Fyfe et al., 2018; Fyfe, Brown, & Coles, 2003; Fyfe, Brown, & Rippon, 2003; Merryfield, 1977; Merryfield & Moore, 1974). This potentially extends to Mesolithic burning and tree clearances (*c*. >6000 BCE) (Fyfe, Brown, & Coles, 2003; Merryfield, 1977; Merryfield & Moore, 1974), and certainly includes disturbances from the Neolithic (*~*4000–500 BCE) and Bronze Age (*~*500 BCE–300 CE) onwards (Fyfe, 2012; Fyfe, Brown, & Coles, 2003). Few pollen studies have examined ecological change since the late Medieval (*~*1400 CE) on Exmoor in such detail, and Figure 6 illustrates that change has been a constant, rather than an exception. While all sites have demonstrably been open moorland for the last 600 years (and probably for at least 2000–4000 years, since the late Neolithic/early Bronze age (Fyfe et al., 2018)), moorland characteristics have not remained unchanged. This is most obvious at Larkbarrow, which has alternated between grass and heather moorland (Callunetum) (see also Chambers et al. (1999)), and at other sites floristic diversity, the relative importance of grasses and *Sphagnum* abundance have also fluctuated through time (Figures 3 and 6). This reflects long‐term moorland responses to burning and grazing practices observed in Scotland, Wales, and Northern England, including reductions in floristic diversity with enhanced grazing pressure (Hanley et al., 2008), and *Sphagnum* declines and graminoid expansion coincident with burning intensification (Chambers et al., 2013; McCarroll et al., 2016). At a community level, rates of change in the last ~600 years were generally greatest during the 19th century, and 19th century rates of change are not distinct from preceding centuries. This suggests that despite landscape‐scale engineering (e.g., drainage) and reorganization (e.g., enclosure) being a feature of 19th century Exmoor (Hegarty & Wilson‐North, 2014; Orwin & Sellick, 1970), with associated changes in vegetation (see above), the pace of vegetation change was not unusual in a post‐medieval context. However, the rates and magnitudes of change among other organismal groups may be different. For example, following drainage at Ricksy Ball substantial turnover in microbial communities (testate amoebae) occurred rapidly, with implications for broader ecosystem productivity (Rowney et al., 2022).

### 4.2 Modern land management and conservation

Paleoecology can be used to provide specific targets for conservation and restoration based on fixed points in the past (i.e., “baselines”) but, in most cases, this is ill‐advised (A. L. Davies & Bunting, 2010). A more pragmatic approach is to use knowledge of the past as “a guide rather than a template” and identify processes that result in particular conditions (Higgs et al., 2014). This develops our understanding of ecosystems and how they may respond to future perturbations and interventions, thereby informing management decisions. Here, we present a range of reference conditions and evidence for the general impacts of various human interventions (processes) on moorland vegetation during the last ~600 years. Even within a relatively small area (*~*60 km^2^), there is no individual “smoking gun” or root cause for the present state of moorland vegetation. Drainage (19th century), burning and grazing are all demonstrably associated with past vegetation changes, including reductions in *Sphagnum*, reductions in taxon richness, increased monocot (graminoid) abundances, and other variables for which sufficiently long‐term data are unavailable (e.g., atmospheric nitrogen deposition) may also be important (Noble et al., 2018). The aims of management interventions intended to increase *Sphagnum* abundances, increase taxon richness and reduce graminoid (e.g., *Molinia caerulea*) dominance (i.e., “restoration” sensu *lato*) are consistent with the long‐term dynamics of Exmoor peatland systems (though site‐specific histories should be borne in mind). Such interventions may include raising water tables through “re‐wetting” to enhance biodiversity and carbon sequestration efficiency (Gatis et al., 2016; Grand‐Clement et al., 2013, 2015; Zak & McInnes, 2022), or alterations to grazing and/or burning regimes (Glaves, 2016). The precise methods used to achieve these aims should be carefully considered with regard to efficacy and societal values (Zak & McInnes, 2022). Furthermore, “restoration” efforts may be considered as a continuation of thousands of years of humans managing moorlands to promote favored ecological outcomes, states, and processes.

## 5 CONCLUSIONS

We demonstrate long‐term associations between multiple anthropogenic disturbances and moorland vegetation. Nineteenth century drainage appears to have led to reductions in *Sphagnum* abundance and vegetation diversity. Over longer timescales, burning is associated with enhanced graminoid monocot abundance and grazing with lower vegetation diversity. Rate‐of‐change in moorland vegetation communities during the 19th century is not distinctive in a long‐term context, and the 20th century is more distinctive in this regard. The aims of “restoration” interventions intended to increase *Sphagnum* abundances, increase taxon richness and reduce graminoid dominance are consistent with the long‐term dynamics of the peatland systems examined here. “Restoration” deemed successful in these terms may or may not resemble pre‐drainage conditions, which were themselves a function of millennia of successive moorland management regimes.

### 5.1 Statement on inclusion

Our research was discussed with local stakeholders during development and disseminated in local outreach activities following completion. The lead institution is approximately 100 km from the study area.

## AUTHOR CONTRIBUTIONS

**Francis Michael Rowney:** Conceptualization (equal); data curation (lead); formal analysis (lead); methodology (equal); writing – original draft (lead); writing – review and editing (lead). **Ralph Fyfe:** Conceptualization (equal); formal analysis (supporting); funding acquisition (equal); methodology (equal); project administration (equal); visualization (supporting); writing – original draft (supporting); writing – review and editing (supporting). **Leonard Baker:** Conceptualization (supporting); data curation (supporting); methodology (supporting); writing – review and editing (supporting). **Henry French:** Conceptualization (supporting); funding acquisition (equal); project administration (equal); writing – review and editing (supporting). **Martha B Koot:** Data curation (supporting); formal analysis (supporting); methodology (supporting); resources (supporting); writing – review and editing (supporting). **Havananda Ombashi:** Data curation (supporting); writing – review and editing (supporting). **Rhys G.O. Timms:** Data curation (supporting); formal analysis (supporting); methodology (supporting); resources (supporting); visualization (supporting); writing – review and editing (supporting).

## CONFLICT OF INTEREST STATEMENT

The authors have no conflicts of interest.

## Supporting information


**Appendix S1.** Supporting Information

## Data Availability

Data are available via the University of Plymouth PEARL repository (https://doi.org/10.24382/0ps2‐6t74) and will also be made available via the European Pollen Database (EPD). https://pearl.plymouth.ac.uk/.
