# Supplementary material for: Historical anthropogenic disturbances explain long‐term moorland vegetation dynamics
Source: Ecol Evol. 2023 Mar 8;13(3):e9876. doi: 10.1002/ece3.9876 (PMC9994610; doi:10.1002/ece3.9876)
Supplement: Supplementary file 1 — Appendix S1. Supporting Information [file ECE3-13-e9876-s001.docx]

**Appendix: Supporting information**

***Methodology***

*Field*

Peat samples for palaeoecological analyses were collected from five sites within the Royal Forest area of Exmoor: Blackpitts (-3.770°, 51.162°), Larkbarrow (-3.687°, 51.171°), Little Ashcombe (-3.749°, 51.148°), Ricksy Ball (-3.808°, 51.131°) and The Chains (-3.813°, 51.164°) (Figure 1). Blackpitts, Larkbarrow and Little Ashcombe were sampled in 2021 using monolith tins for upper sections (*c.* <100 cm) and a closed-chamber corer for lower sections (*c.* >100 cm). Ricksy Ball was sampled followed a similar procedure in 2012 (Rowney et al., 2022), and The Chains was sampled in 2017 using only a closed-chamber corer (Ombashi, 2019). At present, Blackpitts, The Chains and Ricksy Ball are high, open moorland sites with relatively shallow peats (often <1 m) and vegetation dominated by graminoid monocots (*e.g.* *Molinia caerulea*, Cyperaceae), with occasional ericoids (*e.g.* *Calluna vulgaris*, *Vaccinium myrtillus*) and moorland herbs (*e.g.* *Potentilla erecta*). Little Ashcombe is a small area of peat within an enclosed field, with vegetation dominated by graminoids (Poaceae, Cyperaceae) with occasional ruderal herbs (*e.g.* *Plantago lanceolata*, *Cirsium spp.*). Larkbarrow is a small valley mire site adjacent to ‘Larkbarrow Farm’ (historical site) that has vegetation characterised by graminoid monocots (Poaceae, Cyperaceae) with occasional wet acid heath specialists (*e.g.* *Drosera spp.*, *Dactylorhiza praetermissa*).

*Chronology*

Radiocarbon analysis

Samples of peat 1 cm thick were used for AMS (accelerator mass spectrometry) radiocarbon analysis, on the humic acid fraction to avoid contamination by younger carbon from higher levels (Piotrowska, Blaauw, Mauquoy, & Chambers, 2011). There are potential sources of error, but radiocarbon dates of bulk peat are generally reliable (Piotrowska et al., 2011; Väliranta et al., 2014).

Fallout radionuclide (^210^Pb and ^137^Cs)

Contiguous 1 cm samples were taken from the upper parts of the sequences for fallout radionuclide-based sediment dating techniques (^210^Pb and ^137^Cs) (Appleby, 2001), undertaken at the University of Plymouth Consolidated Radioisotope Facility (CORiF, ISO9001:2015 certified). Sediment samples were freeze-dried and homogenised, prior to being packed and sealed into individual 4 mL gas-tight plastic vials. Sample mass was accurately determined using a calibrated balance. Before analysis could start, the sealed samples were incubated for 21 days to allow the development of secular equilibrium within the ^238^U decay chain. Activity concentrations of the target radionuclides (^210^Pb, ^214^Pb, ^137^Cs and ^241^Am) were determined using a High Purity Germanium (HPGe) well-type gamma detector (GWL-170-15-S; N-type), cooled wth liquid nitrogen, and built to ultra-low background specification for detection of ^210^Pb (EG&G Ortec, Wokingham, UK). Based on previous analyses of peat and trial runs of the current samples, it was determined that the samples should be counted for a minimum of 48 hours and that samples with a mass of <2g required a counting time of several days. The gamma spectrometer was calibrated using a sample of natural low-background peat spiked with a certified mixed radioactive standard (#80717-669; Eckert & Ziegler Analytics Georgia, USA) and quantitative spectral analyses were conducted using EG&G GammaVision software. Quality control of instrument performance was conducted by analysis of the IAEA reference Moss Soil (IAEA-CU-2009-03; Vienna, Austria). Total ^210^Pb was determined from its gamma emissions at 46.5 keV and its unsupported component estimated by the subtraction of the ^226^Ra activity, which in turn was determined by the gamma emissions of ^214^Pb at 295 and 352 keV. ^137^Cs was determined from its gamma emission at 662 keV and ^241^Am by its emission at 59.4 keV (Appleby, 2001). All activity concentrations were decay corrected to the date of sampling and uncertainties were derived from counting statistics and reported as 2 *sigma*. The Constant Rate of Supply (CRS) model (Appleby, 2001; Appleby & Oldfield, 1978) as implemented in ‘rplum’ (Aquino-López, Blaauw, Christen, & Sanderson, 2018; Blaauw et al., 2021) was used to construct core chronologies from activity concentrations.

Tephra

Tephra samples were dried overnight (105 °C) (to provide sample dry weights) and combusted at 550 °C for two hours (to remove organic material), before being wet-sieved to retain the 15-125 µm fraction. Volcanic glass shard extraction followed standard guidelines for density separation using Sodium Polytungstate (SPT) (Na_6_ (H_2_W_12_O_40_) H_2_O) (Blockley et al., 2005; Eden, Froggatt, & McIntosh, 1992; Turney, 1998). The specific gravity of the SPT was varied depending on the composition of the samples and the recorded presence of biogenic silica. An initial ‘cleaning float’ designed to remove biogenic silica was applied with a specific gravity ranging from 2.0 to 2.2 cm^-3^. This was followed by an ‘extraction float’ with a specific gravity of 2.55 cm^-3^ designed to preferentially remove silicic volcanic glass shards (Blockley et al., 2005). Extraction residues were rinsed repeatedly with de-ionised water to remove residual SPT before being mounted to glass slides using Canada Balsam. Slides were counted systematically on a polarising light microscope at 200-400x magnification.

Tephra samples were initially taken at contiguous 5 cm intervals. Where peaks of volcanic glass concentrations were identified, corresponding intervals were re-sampled at 1 cm resolution and processed following the methodology outlined above. Resolving the intervals containing tephra to a higher resolution allows for a more accurate pinpointing of primary deposition and hence placement of the tephra isochron. Following higher resolution refinement, peaks in glass shard concentrations were re-sampled and processed as above, with the exception of the combustion stage and mounting in Canada balsam. Instead, residues were retained in water following extraction, pipetted onto well slides and individual glass shards were extracted using a mechanical micromanipulation device fitted with a 5 µl gas chromatography syringe and a 100 µm diameter needle. ‘Picked’ glass shards were transferred to a flat silicon sheet and covered with an epoxy resin to form a ’stub’. Once cured, the resin at the surface of the stub was removed using a series of graded silicon carbide papers and hand polished using a 0.3 µm aluminium oxide solution. The resin stub was carbon coated to improve conductivity and analysed for major and minor elements at the WDS-EPMA (Cameca SX-100) facility at the University of Edinburgh. Probe conditions followed Hayward (2012). Samples were analysed using a beam diameter of 3 µm set at 15 keV, and a current of 0.5 nA for Na and Al, 2 nA for Ca, Fe, K, Si and 60 nA for Mg, P, Ti, Mn. Calibration, precision and drift was assessed by the analysis of internal Lipari and BCR-2G secondary standards.

Data filtering was conducted manually, with inadvertently analysed geological (e.g. quartz) and biological (e.g. phytoliths) materials removed from the dataset. Any volcanic glass analyses totalling less than 93 % were also removed (Hunt & Hill, 1993). The glass chemical data was compared to previously published results from sites across northern Europe. To narrow prospective eruptions, only tephras deposited across this region during the past *c.* 1100 years have been considered (guided by preliminary age-depth models for the sequences).

*Palaeoecology*

Pollen, coprophilous fungal spore and microcharcoal samples were prepared simultaneously (*i.e.* the same samples, preparation and slides were used), using standard pollen extraction methods (Moore et al., 1991). This included disaggregation in potassium hydroxide, cellulose was digested using an acetolysis reaction, before mounting in silicone oil. Acetolysis may degrade Sordaria-type spores, making diagnostic features more difficult to discern (van Asperen, Kirby, & Hunt, 2016), but no such issues were encountered. Known quantities of an exotic markers (*Lycopodium* spores) were added to allow calculation of concentrations and influx rates (Stockmarr, 1971). Typically, three hundred land pollen grains were identified from each sub-sample: a sufficient number for characterising assemblages (Djamali & Cilleros, 2020). Aquatic taxa and fern spores were also counted. Pollen taxonomy broadly follows Bennett (1994) (with some adjustments following peer review). Three fungal spore types demonstrably associated with dung (*Sporormiella*-, *Podospora*- and *Sordaria*-types) (Baker et al., 2013; Perrotti & van Asperen, 2018) were recorded, and their abundances expressed as influx rates (spores cm^-2^ yr^-1^) (Baker et al., 2013; Perrotti & van Asperen, 2018; J. R. Wood & Wilmshurst, 2013). Microscopic charcoal (microcharcoal) abundances were estimated by counting charcoal particles (shards) >50 µm (length), and expressed as influx rates (shards cm^-2^ yr^-1^) (Mooney & Tinner, 2011). Pollen, spores and microcharcoal were recorded on slides simultaneously using high-power (x400-1000) microscopy. For quantitative analyses, pollen abundances are expressed as influx rates (grains cm^-2^ yr^-1^), to ensure that observations are independent of each other and changes in sedimentation rates. Percentages (relative to total land pollen, ~300 per sample) are used for standard pollen diagrams (Figures S7 to S10) and qualitative (descriptive) interpretations of pollen sequences (see below), to aid interpretation of the sequences.

*Archival data*

Human activities (in areas relevant to palaeoecological sampling sites) during Knight family tenure (1818 to 1891) recorded in the ‘Knight family archive’ and other sources (Orwin & Sellick, 1970; Wilson-North, 2017) (see below for archival references) have been summarised into ecologically relevant categories in Table S1. Archival references are as follows:

- 'Papers of the Knight Family of Exmoor Forest', Somerset Heritage Centre, Taunton, A/EJM.
- 'Papers of the Knight Family of Exmoor', Somerset Heritage Centre, Taunton, A/BAZ/1.
- 'Exmoor and Brendon Rentals of the Estate of Frederic Winn Knight, 1864-1886', Devon Heritage Centre, Exeter, 1262M/0/E/20/152-3.

Table S1 Documented activities and summarised categories from the ‘Knight family archive’ (Wilson-North, 2017) and other sources (Orwin & Sellick, 1970)

| **Activity (Original)** | **Activity (Summarised)** |
| --- | --- |
| Sowing Grass | Arable/Fodder Planting |
| Sowing Potatoes |  |
| Planting Turnips |  |
| Planting Rape |  |
| Planting Mangle Wurzle |  |
| Planting Oats |  |
| Planting Winter Wheat |  |
| Sowing New Grass |  |
| Burning | Burning |
| Burning Furze |  |
| Diverting River | Controlling/Creating Waterways |
| Levelling the Bottom of the Waterways |  |
| Cutting Canal |  |
| Diverting the River |  |
| Straightening the Water Course |  |
| Cutting Drains | Drainage |
| Cutting Open Drains |  |
| Deepening Open Drains |  |
| Cutting Drains Beneath Road |  |
| Cutting Drains Beneath Fences |  |
| Cleaning Drains |  |
| Making Culverts |  |
| Drainage |  |
| Repairing Drains |  |
| Spring Tapping and Drainage |  |
| Digging Culvert |  |
| Drainage (Tom's Hill) |  |
| Drainage (Larkbarrow) |  |
| Repairing and Modernising Drainage (Larkbarrow) |  |
| Cutting New Drainage |  |
| Pasturing Cattle | Grazing |
| Pasturing Sheep (Summering) |  |
| Pasturing Cattle (Summering) |  |
| Pasturing Horses |  |
| Pasturing Sheep |  |
| Pasturing Stock |  |
| Preparing New Allotment for Cattle |  |
| Preparing New Allotment for Sheep |  |
| Increasing Sheep Stock |  |
| Lambing |  |
| Pasturing Cattle (Larkbarrow) |  |
| Increase Sheep Flock |  |
| Pasturing Sheep (Tom's Hill) |  |
| Irrigation (Water Carriage) | Irrigation |
| Cutting Gutters |  |
| Spring Tapping |  |
| Irrigation |  |
| Floating Gutters (Water Meadows) |  |
| Irrigation (Tom's Hill) |  |
| Cutting Water-Meadows (Tom's Hill) |  |
| Irrigation (Larkbarrow) |  |
| Cutting Water-Meadows |  |
| Spading | Levelling/Spading |
| Levelling Land |  |
| Spading Land |  |
| Levelling Land (Meadows) |  |
| Liming | Liming/Fertilisation |
| Spreading Superphosphate of Lime |  |
| Liming Fields |  |
| Spreading Superphostphate of Lime |  |
| Spreading Nitrate of Soda |  |
| Exterminating Wildlife | Miscellaneous |
| Cutting Furze |  |
| Planting New Hedges | Tree/Hedge Planting |
| Banking the Lower Marsh |  |
| Creating Banks |  |
| Planting Trees |  |
| Replant Beech Tree Plantation |  |
| Turf Cutting | Turf Cutting |
| Cutting Turf |  |

*Dataset preparation*

For meta-analyses (rate-of-change, cluster, ordination and regression analyses), pollen and spore data from each site were combined into a single dataset (n=244 before filtering; n=208 final dataset). Taxonomies were standardised (Bennett, 1994) and samples older than 1400 CE were removed (18 samples, ~8 % of n=244) (these are mostly confined to one site: Ricksy Ball). As summer hydroclimate is a key control on peatland water tables (Charman, 2007), annually reconstructed summer precipitation for England and Wales 1201-2000 CE (based on stable oxygen isotopes in tree rings (Loader et al., 2020)) was added as a control variable, requiring the removal of samples younger than 2000 CE (14 samples, ~6 % of n=226). Single-year values and multi-year moving averages for precipitation reconstructions were both tested, with negligible differences between model outcomes. For analyses not requiring a climate control (cluster, multivariate and rate-of-change analyses), these samples were not removed and a slightly larger dataset (n=226) was used. In the following analyses influx rates (grains/spores cm^-2^ yr^-1^) were used to ensure observations are independent and changes in sedimentation rates are accounted for.

*Cluster analysis*

Cluster analysis was used with rate-of-change analysis to classify groups of samples with similar palaeoecological assemblages and to identify clusters associated with periods of rapid ecological change. The distance matrix was created (with the ‘vegdist’ R function in ‘vegan’ (Oksanen et al., 2022)), using Bray-Curtis dissimilarity (Bray & Curtis, 1957): a robust and widely-applied metric for assessing compositional dissimilarity. Clusters were generated from the distance matrix using a complete linkage approach (‘hclust’ R function (R Core Team, 2022)) (there were minimal differences between linkage methods). The approach has been successfully applied on pollen compositional data to explore natural groupings within large datasets (Fyfe, Woodbridge, & Roberts, 2018). Five clusters were used here, corresponding to the number of sites, to allow for the potential that spatial clustering may be more important than temporal.

*Rate-of-change analysis*

Rate-of-change analysis was used to explore the pace of ecological change through time, and in particular whether nineteenth century rates-of-change are notable in a long-term context (*~*600 years). We used an approach based on measures of dissimilarity between ‘bins’ of pollen samples (influx rates) within ‘moving windows’, using the R package ‘R-Ratepol’ (Mottl et al., 2021). Euclidean distances were used, as these are appropriate for assessing differences between compositionally-similar assemblages (*e.g.* adjacent pollen samples), as recommended for pollen influx rates (Mottl et al., 2021). Assemblage data were smoothed using age-weighted averages, to account for varying sample resolution within sequences (Mottl et al., 2021). The maximum time represented by a sample (according to age-depth modelling) was 25.6 years, and so a bin size of 26 years was chosen, with four ‘shifts’, resulting in rate-of-change estimates at six-year resolution.

*Regression modelling*

*Sphagnum* and monocot influx rates were both ‘heavy-tailed’, and scaled t-distributions were used for these. Non-arboreal taxon richness followed a Gaussian distribution. The dataset is not large (n=208) and using as few explanatory variables as possible is sensible to avoid overfitting. Accordingly, ‘select=TRUE’ was used, introducing automatic variable selection (variables that have ‘no effect’ are penalised to zero). The R code followed a general form, outlined here:

mgcv::gam(Sphagnum ~ s(SummerPrecipitation, bs="ts") + s(CoprophilousFungi, bs="ts") + s(Microcharcoal, bs="ts") + Drainage + SiteType + s(Site, bs="re"), data=pollen, family="scat", select=TRUE, method="REML")

***Results***

*Descriptive statistics*

Table S2 Summary statistics for continuous and categorical variables used in regression modelling (n=208)

| **Continuous variables** | | **n** | **Mean** | | | **Median** | **Min** | **Max** | **SD** |
| --- | --- | --- | --- | --- | --- | --- | --- | --- | --- |
| *Sphagnum* influx  (spores cm^-2^ yr^-1^) | | 208 | 560.66 | | | 276.61 | 0.00 | 5370.98 | 835.52 |
| Graminoid monocot influx  (grains cm^-2^ yr^-1^) | | 208 | 3013.95 | | | 1990.24 | 227.53 | 14977.27 | 2763.02 |
| Non-arboreal pollen richness | | 208 | 13.37 | | | 14.00 | 4.00 | 21.00 | 3.63 |
| Summer precipitation | | 208 | 297.59 | | | 301.55 | 122.50 | 480.20 | 68.02 |
| Coprophilous fungal spore influx  (spores cm^-2^ yr^-1^) | | 208 | 72.98 | | | 42.31 | 0.00 | 433.88 | 84.81 |
| Microcharcoal (>50 µm) influx  (shards cm^-2^ yr^-1^) | | 208 | 883.65 | | | 357.14 | 0.00 | 11138.55 | 1432.84 |
|  | |  |  | | |  |  |  |  |
| **Categorical variables** | **Category** | | | **n** |  |  |  |  |  |
| Site | Blackpitts | | | 42 |  |  |  |  |  |
|  | Larkbarrow | | | 45 |  |  |  |  |  |
|  | Little Ashcombe | | | 34 |  |  |  |  |  |
|  | Ricksy Ball | | | 27 |  |  |  |  |  |
|  | The Chains | | | 60 |  |  |  |  |  |
| Site type | Ombrotrophic | | | 129 |  |  |  |  |  |
|  | Soligenous | | | 79 |  |  |  |  |  |
| Drainage | Pre-drainage | | | 129 |  |  |  |  |  |
|  | Post-drainage | | | 79 |  |  |  |  |  |

*Chronology*

The following relates to The Chains, Blackpitts, Larkbarrow and Little Ashcombe. Chronological details for Ricksy Ball have been published (Rowney et al., 2022).

Radiocarbon

Thirty samples were processed for radiocarbon dating for Blackpitts (thirteen), The Chains (six), Larkbarrow (five) and Little Ashcombe (six) (Table S3). Radiocarbon dates of bulk peat are generally reliable, though there are a number of potential sources of error (Piotrowska et al., 2011; Väliranta et al., 2014).

Table S3 Calibrated radiocarbon (^14^C) dates before and after age-depth modelling (minimum and maximum represent 95 % confidence intervals). Radiocarbon dates calibrated using IntCal20 (Reimer et al., 2020). Modelled dates are based on age-depth modelling. (Samples marked with * previously available in Ombashi (2019).)

| **Site** | **Sample** | **Depth (cm)** | **Uncal. ^14^C Age (^14^C yr)** | **Calibrated Age**  **(cal BP)** | | | **Modelled Age**  **(cal BP)** | | |
| --- | --- | --- | --- | --- | --- | --- | --- | --- | --- |
|  |  |  |  | Min | Max | Prob | Mean | Min | Max |
| **Blackpitts** | UBA-44914 | 20 | -87 | Invalid for IntCal20 | | | 39 | 19 | 57.4 |
|  | UBA-45691 | 25 | 405 | 512  435  359 | 438  433  330 | 80.1  0.5  14.3 | 98 | 83 | 144 |
|  | UBA-47327 | 26 | 269 | 425  385  318  164 | 391  380  287  157 | 27.2  1.3  62.4  3.8 | 114 | 94 | 162 |
|  | UBA-47328 | 27 | 58 | Invalid for IntCal20 | | | 133 | 115 | 175 |
|  | UBA-47330 | 28 | 229 | 309  274  208  185  173  8 | 277  273  200  177  152  0 | 47.8  0.4  2.3  3  35.2  6.2 | 159 | 141 | 187 |
|  | UBA-44915 | 30 | 207 | 301  215  190  21  14 | 267  194  148  16  0 | 27.9  12  40.5  1.7  12.7 | 190 | 161 | 217 |
|  | UBA-47331 | 32 | 73 | Invalid for IntCal20 | | | 219 | 183 | 248 |
|  | UBA-47332 | 33 | 127 | 266  194  148  18 | 214  190  20  12 | 24.1  1.2  68.1  1.5 | 230 | 192 | 262 |
|  | UBA-47333 | 34 | 166 | 284  225  155  113  101  80  35 | 255  166  139  103  82  72  0 | 17.2  39.7  9.8  2.1  3.6  1.8  20.8 | 240 | 206 | 274 |
|  | UBA-45692 | 35 | 19 | Invalid for IntCal20 | | | 253 | 223 | 288 |
|  | UBA-44916 | 40 | 264 | 423  317  165 | 398  286  156 | 16.6  71.9  6.4 | 327 | 291 | 415 |
|  | UBA-44917 | 60 | 743 | 713  689 | 713  663 | 0.3  94.6 | 694 | 665 | 734 |
|  | UBA-44918 | 80 | 854 | 788  703 | 723  697 | 93.2  1.8 | 1049 | 979 | 1140 |
| **Larkbarrow** | UBA-45685 | 20.5 | 211 | 303  216  191  22 | 267  194  147  0 | 29.9  11.6  39.5  13.9 | 237 | 196 | 273 |
|  | UBA-45686 | 30.5 | 487 | 543 | 499 | 94.6 | 534 | 493 | 623 |
|  | UBA-45687 | 40.5 | 1070 | 1056  1005 | 1023  926 | 23.2  71.7 | 928 | 805 | 1006 |
|  | UBA-45688 | 60.5 | 1657 | 1689  1611  1499  1490  1454 | 1673  1510  1495  1472  1416 | 5.9  71.3  0.7  5.4  11.5 | 1467 | 1387 | 1571 |
|  | UBA-45689 | 93.5 | 2182 | 2314  2078 | 2101  2072 | 94.3  0.7 | 2171 | 2036 | 2298 |
| **Little Ashcombe** | UBA-45679 | 20.5 | 517 | 621  554 | 616  507 | 1.8  93 | 400 | 307.7 | 543.7 |
|  | UBA-45680 | 30.5 | 893 | 904  856 | 866  847 | 24.5  2.3 | 772.2 | 708.7 | 871.1 |
|  | UBA-45681 | 40.5 | 1223 | 1262  1248  1177  1171 | 1253  1209  1173  1065 | 2  17.3  2.4  73.2 | 1088.7 | 999.2 | 1159.6 |
|  | UBA-45682 | 60.5 | 1764 | 1715  1681 | 1683  1587 | 22.4  72.5 | 1616.9 | 1542.3 | 1699.3 |
|  | UBA-45683 | 80.5 | 2979 | 3321  3246  3036  3011 | 3304  3062  3036  3008 | 2.1  92.5  0.1  0.3 | 1897.8 | 2181.5 | 2029.6 |
|  | UBA-45684 | 126.5 | 2796 | 2989  2963  2825 | 2987  2844  2789 | 0.4  87.4  7.2 | 2911.1 | 2803.6 | 3040.5 |
| **The Chains** | UBA-45690 | 21 | 108 | 263  143 | 220  24 | 25.2  69.8 | 198 | 73 | 275 |
|  | UBA-44909 | 25 | 235 | 418  313  209  187  10 | 414  270  198  151  0 | 0.7  51.8  2.9  33.8  5.8 | 256 | 142 | 323 |
|  | UBA-44910 | 30 | 269 | 427  326  166 | 375  285  156 | 34.5  54.8  5.6 | 341 | 280 | 429 |
|  | UBA-44911 | 35 | 318 | 457  342 | 348  308 | 74.4  20.6 | 405 | 316 | 478 |
|  | UBA-44912 | 40 | 457 | 536 | 477 | 94.8 | 499 | 426 | 539 |
|  | UBA-44913 | 45 | 550 | 626  558 | 595  524 | 33.3  61.4 | 540 | 507 | 578 |
|  | UBA-38053* | 60.5 | 498 | 547 | 503 | 94.7 | 619 | 559 | 665 |
|  | UBA-38054* | 120.5 | 1249 | 1281  1169 | 1175  1069 | 53.4  41.5 | 1193 | 1072 | 1287 |
|  | UBA-38055* | 180.5 | 1868 | 1868  1839 | 1853  1709 | 3.8  91.2 | 1833 | 1722 | 2016 |
|  | UBA-38056* | 220.5 | 2715 | 2875 | 2755 | 94.9 | 2621 | 2189 | 2858 |

Fallout radionuclide (^210^Pb and ^137^Cs)

Activity concentrations for ^210^Pb and ^137^Cs from Blackpitts, Larkbarrow and Little Ashcombe are given in Table S4. Measurements did not reach background levels in the Little Ashcombe samples, but ‘rplum’ (Blaauw et al., 2021) has been demonstrated to handle ‘missing’ data (Aquino-López et al., 2018). Measurement of ^210^Pb and ^137^Cs for The Chains was unsuccessful, due to limited material available for dating.

Table S4 Activity concentrations for ^210^Pb and ^137^Cs. (MDA = Minimum Detectable Activity)

| ***Site*** | ***Depth (cm)*** | ***Bq kg^-1^*** | | | | ***Detector Run Days*** |
| --- | --- | --- | --- | --- | --- | --- |
|  |  | ***^137^Cs*** | ***Error (2σ)*** | ***^210^Pb*** | ***Error (2σ)*** |  |
| **Blackpitts** | 1 | 34.8 | 10.7 | 399 | 71 | 3 |
|  | 3 | 63.4 | 19.7 | 680 | 123 | 2 |
|  | 4 | 63.6 | 19 | 636 | 117 | 2 |
|  | 5 | 42.3 | 9.5 | 465 | 62 | 3 |
|  | 6 | 23.5 | 8.6 | 247 | 60 | 3 |
|  | 7 | 52.7 | 13.2 | 371 | 76 | 3 |
|  | 8 | 36.7 | 11.2 | 275 | 58 | 2 |
|  | 9 | 55.5 | 14.9 | 388 | 98 | 2 |
|  | 10 | 56.8 | 10.7 | 350 | 64 | 2 |
|  | 11 | 52.4 | 7.5 | 329 | 44 | 2 |
|  | 12 | 47.8 | 8.1 | 388 | 50 | 2 |
|  | 13 | 51.6 | 9.2 | 387 | 56 | 2 |
|  | 14 | 42.5 | 6.5 | 407 | 50 | 2 |
|  | 15 | 32.5 | 7.6 | 306 | 52 | 2 |
|  | 16 | 27.7 | 6.4 | 191 | 35 | 2 |
|  | 17 | 21 | 8.1 | 138 | 34 | 1 |
|  | 18 | 12.3 | 6.3 | 88.4 | 31 | 2 |
|  | 19 | 20.4 | 9 | 44.7 | MDA | 1 |
|  | 20 | 11.47 | 3.67 | 73.74 | 24.7 | 3 |
| **Larkbarrow** | 1 | 26.06 | 5.28 | 369.65 | 58.18 | 4 |
|  | 2 | 34.64 | 8.84 | 551.11 | 95.38 | 2 |
|  | 3 | 55.56 | 10.23 | 548.05 | 77.67 | 2 |
|  | 4 | 56.03 | 10.96 | 329.03 | 62.5 | 2 |
|  | 5 | 30.84 | 7.97 | 245.75 | 54.44 | 2 |
|  | 6 | 26.27 | 4.84 | 180.46 | 36.41 | 4 |
|  | 7 | 20.49 | 4.05 | 177.63 | 46.1 | 2 |
|  | 8 | 19.12 | 5.81 | 103.69 | 39.65 | 2 |
|  | 9 | 19.68 | 5.12 | 65.75 | 30.28 | 3 |
|  | 10 | 16.84 | 5.08 | 54.49 | 32.53 | 3 |
|  | 11 | 16.61 | 3.98 | 50.01 | 21.21 | 2 |
|  | 12 | 19.06 | 5.1 | 25.76 | MDA | 3 |
|  | 13 | 17.85 | 4.4 | 21.64 | MDA | 4 |
|  | 14 | 17.01 | 5.22 | 23.53 | MDA | 3 |
|  | 15 | 17.08 | 5.09 | 24.24 | MDA | 3 |
|  | 16 | 10.48 | 3.86 | 21.52 | MDA | 3 |
|  | 17 | 12.38 | 5.16 | 28.87 | MDA | 3 |
|  | 18 | 10.51 | 4.69 | 24.88 | MDA | 3 |
|  | 19 | 7.56 | 3.47 | 24.32 | MDA | 3 |
|  | 20 | 9.93 | 4.18 | 21.07 | MDA | 3 |
| **Little Ashcombe** | 1 | 23.57 | 9.08 | 403 | 80.2 | 3 |
|  | 2 | 14.27 | 3.80 | 292.9 | 59.2 | 3 |
|  | 3 | 17.64 | 9.16 | 306 | 83.8 | 3 |
|  | 4 | 23.98 | 9.24 | 217.37 | 71.68 | 4 |
|  | 5 | 11.98 | 7.26 | 263.03 | 48.50 | 3 |
|  | 6 | 23.70 | 7.89 | 203.33 | 70.88 | 4 |
|  | 7 | 17.68 | 5 | 215 | 56.0 | 3 |
|  | 8 | 25.84 | 9.73 | 294 | 75.8 | 3 |
|  | 9 | 25.88 | 9.04 | 243 | 59.9 | 3 |
|  | 10 | 27.07 | 6.64 | 256 | 51.0 | 4 |

Tephra

Six cryptotephra horizons across the three sites were chosen for chemical analysis: three in Blackpitts, two in Little Ashcombe and one in Larkbarrow. Several chemical populations are present in each analysed interval (Figure S1). Five of these were assigned ages based on geochemical similarities to tephras from known eruptions or independently-dated cryptotephra horizons in other sites/regions (see Table S5 and Figure S2). The known ages of these tephras were used in the construction of site-specific age-depth models. In some cases (*e.g.* LAM1 18-19), a single tephra could still not be determined, and an age range was assigned based on several potential dates. One tephra horizon (Larkbarrow, 23-24 cm) was not confidently identified and therefore not included in age-depth models.

A summary of tephra correlations is presented in Table S5. The ages for the majority of the tephras identified are well documented in historical texts (e.g. see Larsen et al. 1999; Plunket and Pilcher 2018 and references therein). The MOR-T4 Tephra has previously been assigned a date of *c.* 1000 CE based on linear interpolation between two other tephras (Chambers, Daniell, Hunt, Molloy, & O’Connell, 2004; Holmes, Leuenberger, Molloy, & O’Connell, 2020). Here we adopt a pragmatic approach and assign an age range for MOR-T4 based on the tephras above (Hekla 1104) and below (CBR-90, *c.* 1030 CE) in Holmes et al. (2020): 1067±37 CE (*i.e.* 1030 to 1104 CE). It has previously been suggested that Iceland was the source of MOR-T4 (Chambers et al., 2004), but recent work has suggested Mexico (Jala Pumice) as a potential source area (Plunkett & Pilcher, 2018), raising the possibility of ultra-distal tephras in southwest England.


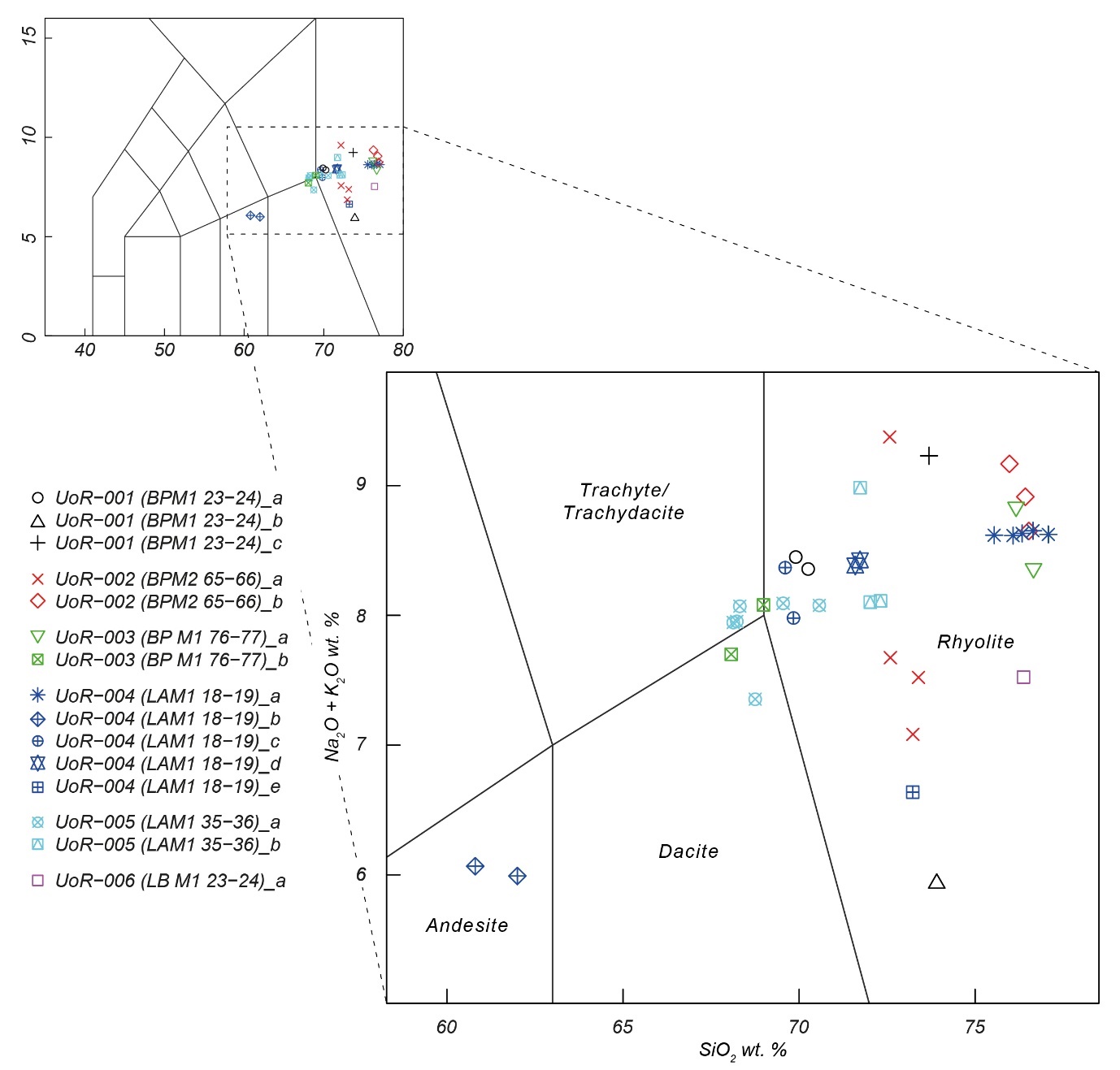


Figure S1 Summary of tephra geochemical classifications

Table S5 Summary of tephra identifications and assigned ages

| **Site** | **Depth**  **(cm)** | **Lab code** | **Chemical population** | **Chemical**  **stub code** | **Identification** | **Age (CE)** | **Age (BP)** |
| --- | --- | --- | --- | --- | --- | --- | --- |
| Blackpitts | 23-24 | BPM1 23-24 | b | UoR-001 | Askja 1875 | 1875 | 75 |
|  | 65-66 | BPM2 65-66 | a | UoR-002 | Hekla 1104 | 1104 | 846 |
|  | 76-77 | BPM1 76-77 | b | UoR-003 | MOR-T4* | 1067±37 | 883±37 |
| Larkbarrow | 23-24 | LBM1 23-24 | a | UoR-006 | Indeterminate | N/A | N/A |
| Little  Ashcombe | 18-19 | LAM1 18-19 | b, c, e | UoR-004 | Hekla 1845  Katla *c.* 1860  Askja 1875 | 1860±15 | 90±15 |
|  | 35-36 | LAM1 35-36 | a | UoR-005 | MOR-T4* | 1067±37 | 883±37 |

*(Holmes et al., 2020)


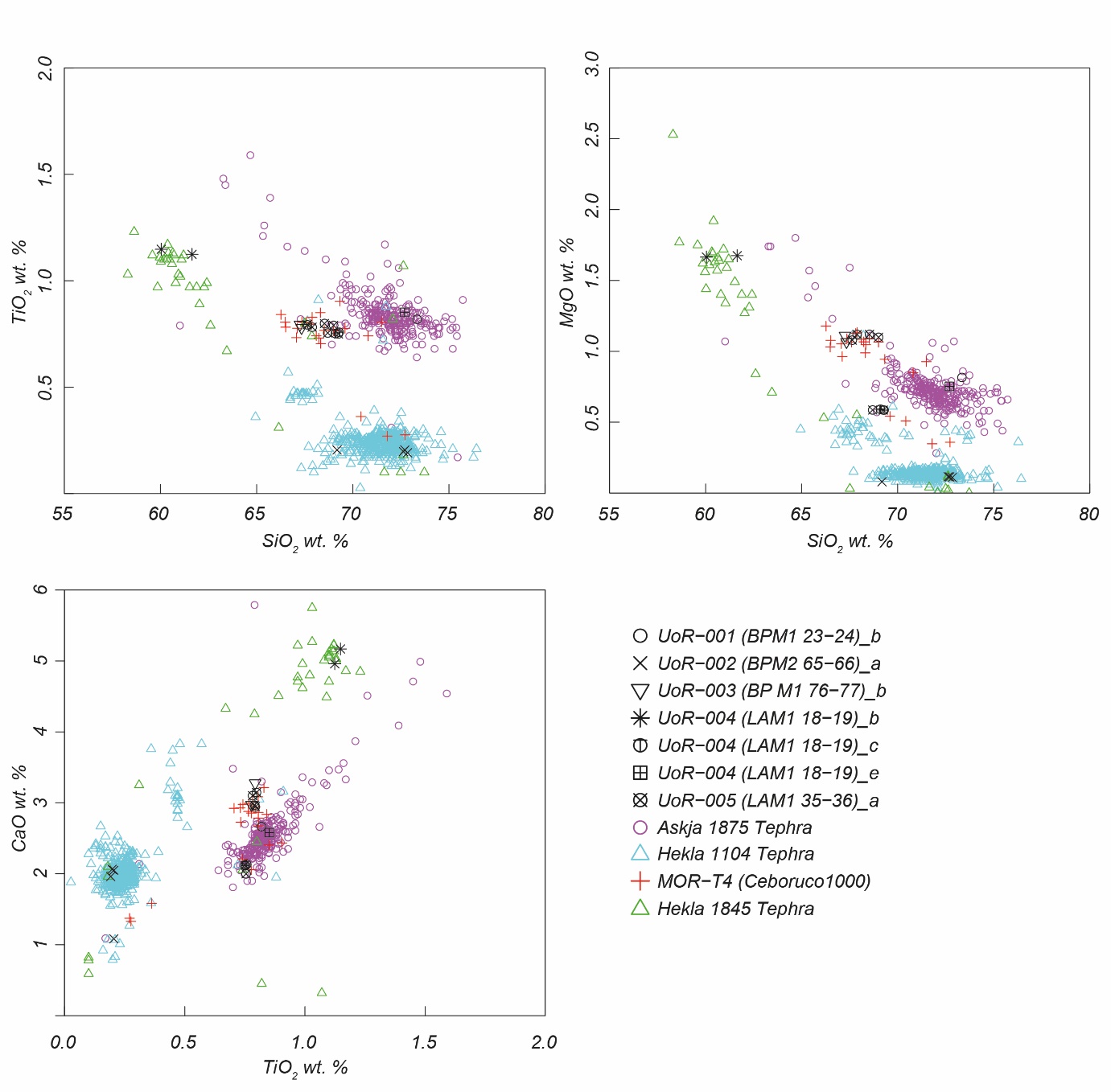


Figure S2 Comparison of tephra geochemical characteristics with known eruptions

Age-depth modelling

The following figures (Figures S3 to S6) provide summaries of age-depth models for Blackpitts, The Chains, Larkbarrow and Little Ashcombe. The age-depth model for Ricksy Ball has been published previously (Rowney et al., 2022).


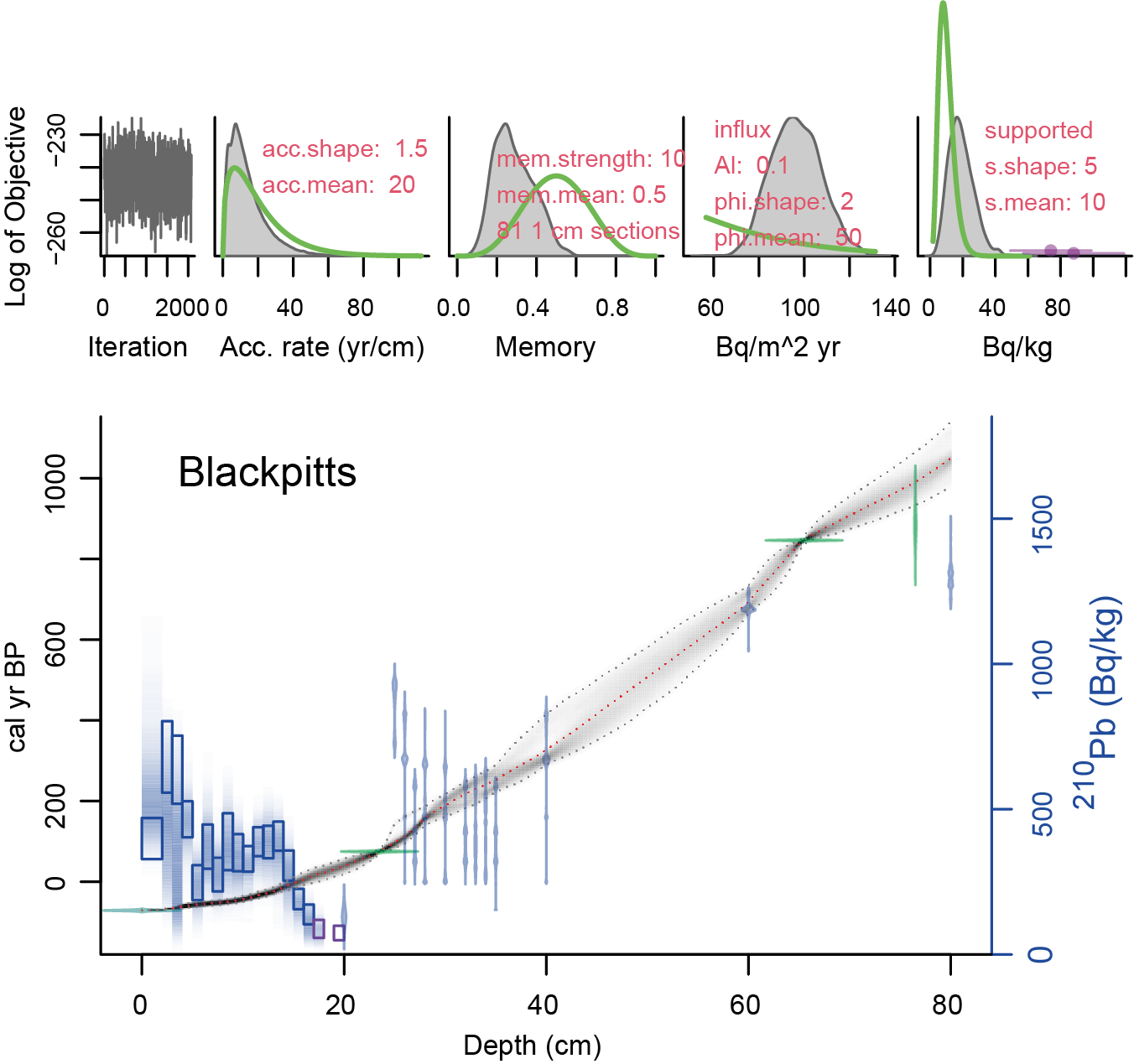


Figure S3 Age-depth model for Blackpitts, based on radiocarbon (blue), tephra (green) and ^210^Pb (boxes). Model constructed using the R package ‘rplum’ (Blaauw et al., 2021)


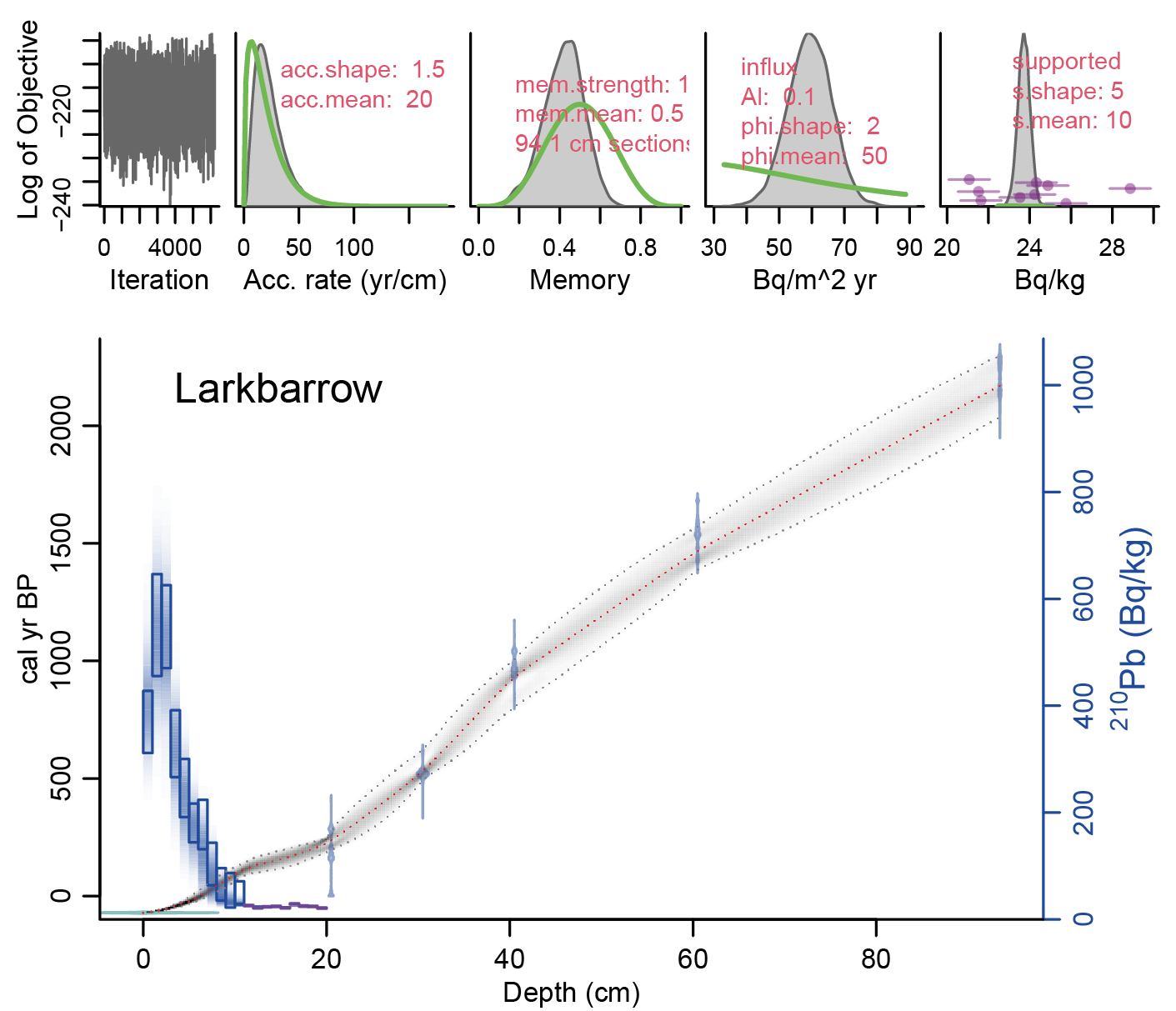


Figure S4 Age-depth model for Larkbarrow, based on radiocarbon (blue) and ^210^Pb (boxes). Model constructed using the R package ‘rplum’ (Blaauw et al., 2021)


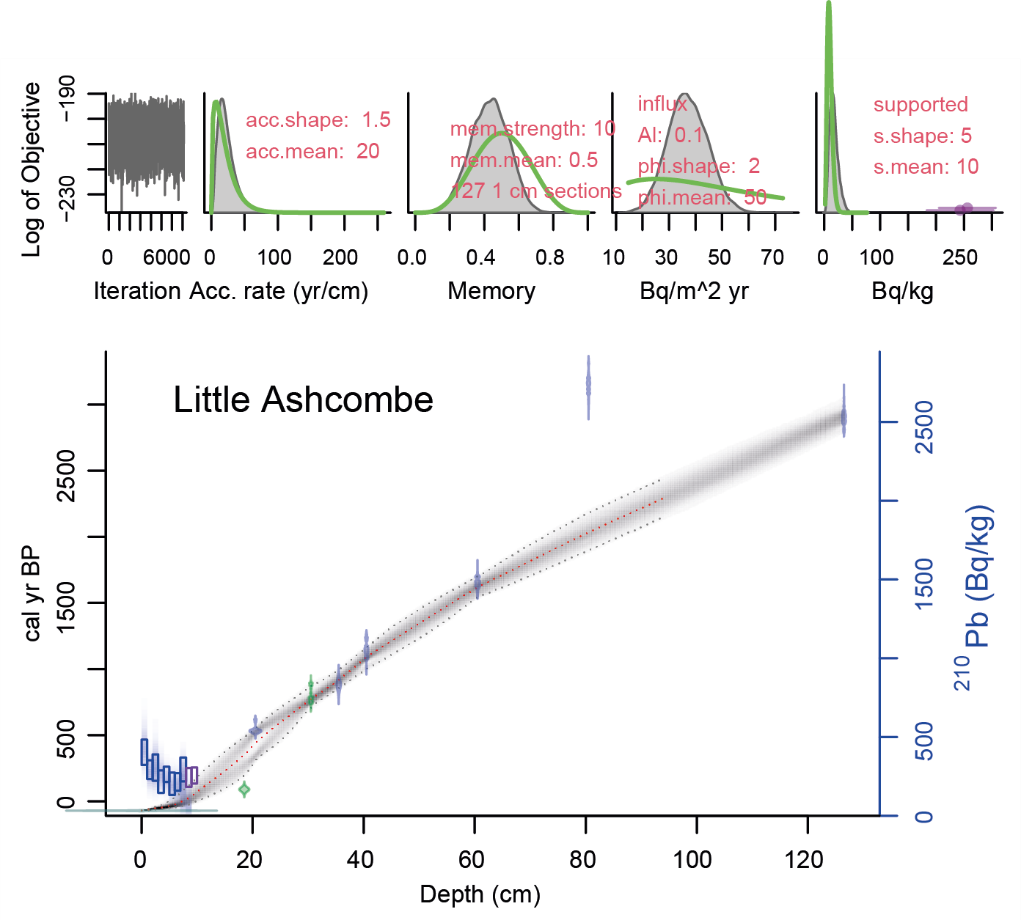


Figure S5 Age-depth model for Little Ashcombe, based on radiocarbon (blue), tephra (green) and ^210^Pb (boxes). Model constructed using the R package ‘‘rplum’ (Blaauw et al., 2021)


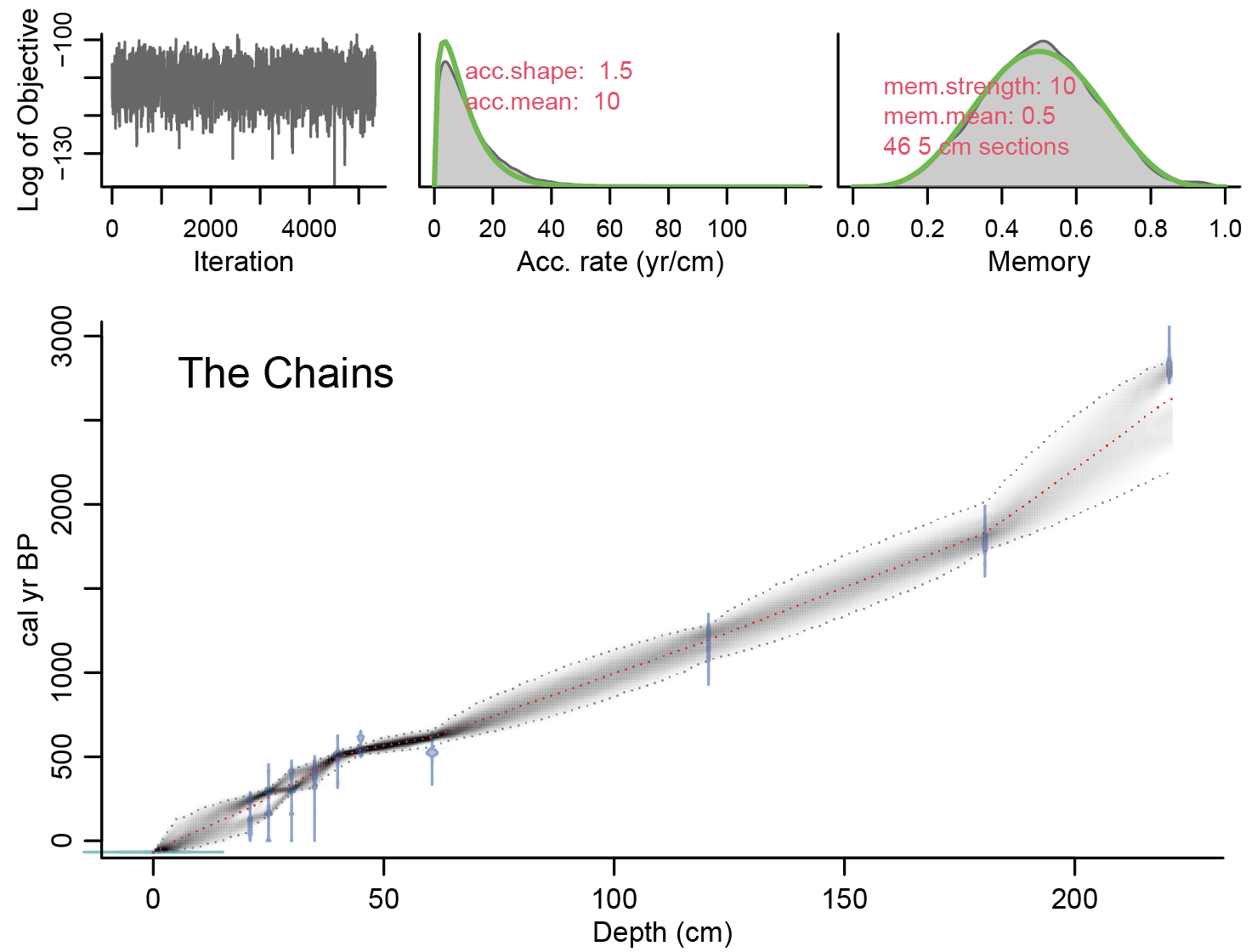


Figure S6 Age-depth model for The Chains, based on radiocarbon. Model constructed using the R package ‘rbacon’ (Blaauw et al., 2022)

*Palaeoecology*

Pollen and spore sequences are summarised below, with abundances primarily expressed as percentages, except for fungal spores and microcharcoal abundances, which are expressed as influx values (spores/shards cm^-2^ year^-1^). Whilst quantitative analyses involving pollen data in the main body of this paper are based on influx values (relevant data are shown in Figure 3), it is important to also provide a more ‘traditional’ summary of the data using percentages, to aid interpretation of the sequences. Pollen zones are determined by CONISS and a broken stick model using the R package ‘rioja’ (Juggins, 2020), and are based on pollen percentages. Pollen data from Ricksy Ball has been previously published (Rowney et al., 2022).

Blackpitts

The pollen data for Blackpitts (Figure S7) demonstrates that the site has been open moorland for at least the last ~600 years, with a high proportion of monocots (Poaceae and Cyperaceae), and some heather (*Calluna vulgaris*) and herbaceous taxa (*e.g.* *Plantago lanceolata*, *Potentilla*-type). Tree and shrub pollen (*e.g.* *Betula*, *Corylus avellana*-type, *Quercus*) is also present, likely drawn from the surrounding landscape (>100 m). During the late nineteenth century, the moorland at Blackpitts appears to have become more monocot-dominated, with decreased heather abundance.


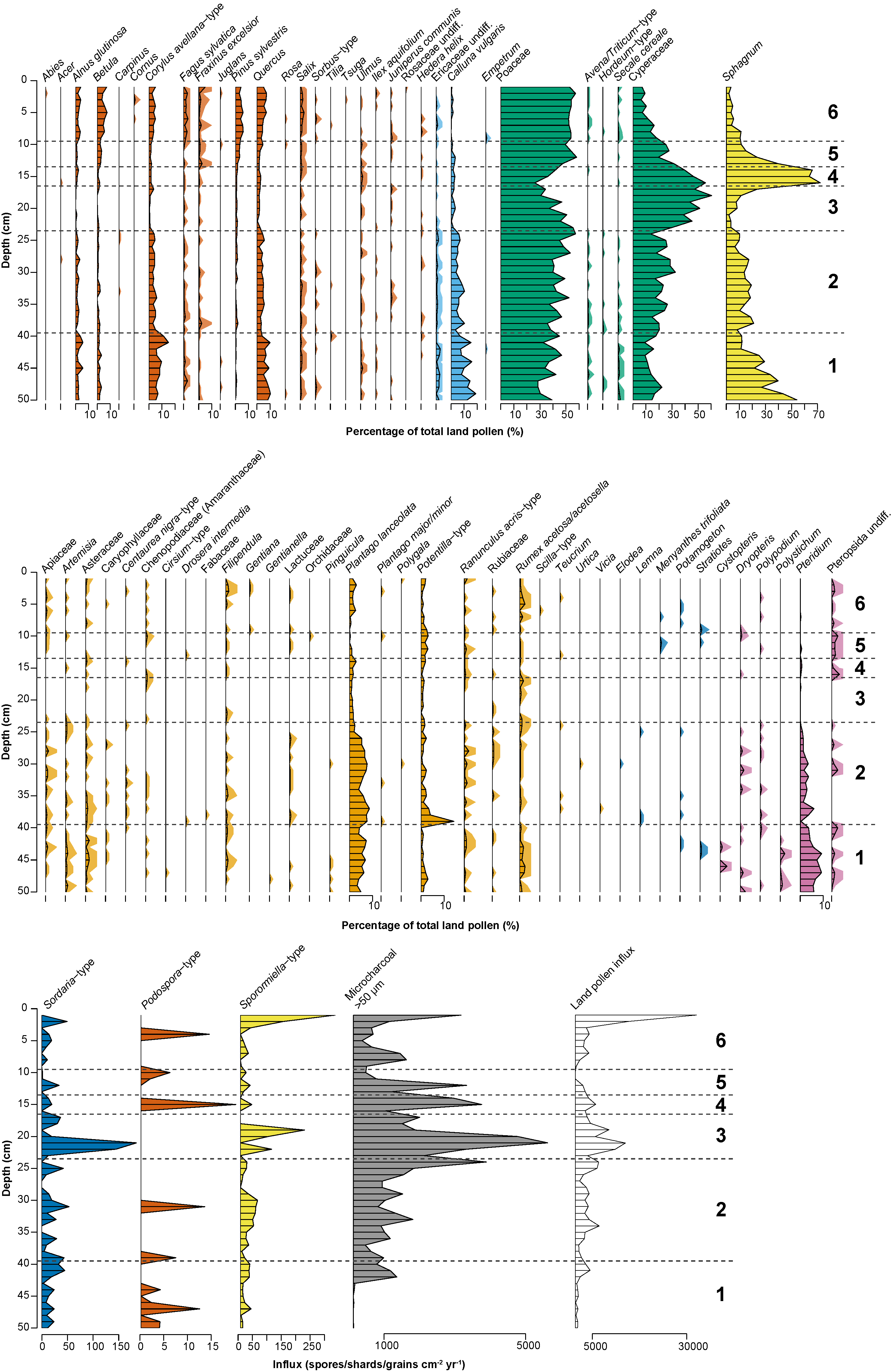


Figure S7 Summary of Blackpitts pollen record. Numbers in bold refer to pollen zones objectively determined by CONISS (Grimm, 1987; Juggins, 2020)

In Blackpitts Zone 1 (50 to 39 cm, *c.* 507 to 311 cal BP / 1443 to 1639 CE), there is a relatively high proportion of trees and shrubs (23.30 % mean), largely represented by *Corylus avellana*-type and *Quercus*. Heathers are at their highest values in the sequence (11.80 % mean *Calluna vulgaris*) and there are moderate quantities of Poaceae (36.90 % mean) and Cyperaceae (14.60 % mean). Herbaceous pollen (10.34 % mean) is largely comprised of *Plantago lanceolata* (5.32 % mean), with some *Potentilla*-type (1.12 % mean) and *Rumex acetosa/acetosella* (1.03 % mean). *Sphagnum* abundance is relatively high (28.10 % mean), but declining. *Sordaria*-, *Podospora*- and *Sporormiella*-type spores are all present (39.08 spores cm^-2^ yr^-1^ mean). Evidence of burning (microcharcoal >50 µm) is largely absent until 42 cm (*c.* 361 cal BP / 1589 CE).

In Blackpitts Zone 2 (39 to 23 cm, *c.* 311 to 68 cal BP / 1639 to 1882 CE), there are decreases in the relative abundances of tree and shrub (14.19 % mean) and *Calluna vulgaris* (6.08 % mean). Both Poaceae (44.30 % mean) and Cyperaceae (22.40 % mean) pollen increase. There is a brief spike in *Potentilla*-type pollen at 39 cm (14.29 %), but otherwise herbaceous assemblages are largely similar (collectively, 11.50 % mean). *Sphagnum* relative abundance is lower than the previous zone (14.10 % mean), but relatively stable. *Sordaria*-, *Podospora*- and *Sporormiella*-type spores continue to be present (44.32 spores cm^-2^ yr^-1^ mean), though *Podospora*-type becomes less common (two occurrences). Evidence of burning (microcharcoal >50 µm) continues (1186.94 shards cm^-2^ yr^-1^ mean), increasing to 3736.63 shards cm^-2^ yr^-1^ at 24 cm (*c.* 82 cal BP / 1868 CE).

Blackpitts Zone 3 (23 to 16 cm, *c.* 68 to 6 cal BP / 1882 to 1944 CE) has the lowest tree and shrub pollen abundances for this site (6.54 % mean). *Calluna vulgaris* pollen also notably decreases (1.76 % mean). Poaceae relative abundances (42.92 % mean) generally decrease in this zone, whilst Cyperaceae (45.22 % mean) is increases, leading to higher monocot abundances overall. The lowest relative abundances of herbaceous pollen in the sequence are recorded in this zone (3.37 %). *Sphagnum* is at low abundance (8.60 % mean), reaching the minimum value (1.99 %) for this site at 21 cm (*c.* 49 cal BP / 1901 CE). Coprophilous fungal spore influx is higher than in the previous zone (121.27 spores cm^-2^ yr^-1^ mean) suggesting increased grazing intensity, although *Podospora*-type is absent. Microcharcoal (>50 µm) influx is higher on average than the previous zone (2895.00 shards cm^-2^ yr^-1^ mean), peaking at 21 cm (*c.* 49 cal BP / 1901 CE).

Blackpitts Zone 4 (16 to 13 cm, *c.* 6 to -24 cal BP / 1944 to 1974 CE) is primarily distinguished from the previous zone by *Sphagnum* relative abundance (67.16 % mean). Poaceae abundance is also relatively low (34.66 % mean), whilst Cyperaceae is relatively high (47.21 % mean). Coprophilous fungal spore influx (30.65 spores cm^-2^ yr^-1^ mean) is also notably lower than the previous zone (including absence at 16 cm, *c.* 6 cal BP / 1944 CE), indicating reduced grazing intensity. Evidence of burning (microcharcoal >50 µm) remains present (2453.81 shards cm^-2^ yr^-1^ mean).

Blackpitts Zone 5 (13 to 9 cm, *c.* -24 to -48 cal BP / 1944 to 1998 CE) is marked by decreasing *Sphagnum* (22.47 % mean) and Cyperaceae (26.02 % mean), whilst Poaceae increases (52.17 % mean). Tree and shrub pollen relative abundances increase in this zone (14.55 %). Herbaceous pollen also remains low (5.04 % mean), with *Plantago lanceolata* absent at 11 cm (*c.* -37 cal BP / 1987 CE). Coprophilous fungal spore influx (27.99 spores cm^-2^ yr^-1^ mean) remains low, suggesting little change in local grazing intensity. Evidence of burning (microcharcoal >50 µm) remains present (1334.60 shards cm^-2^ yr^-1^ mean).

In Blackpitts Zone 6 (9 to 0 cm, *c.* -48 to -71 cal BP / 1998 to 2021 CE), trees and shrubs increase (27.94 % mean), driven by a combination of taxa including *Alnus glutinosa*, *Betula*, *Corylus avellana*-type, *Fagus sylvatica*, *Pinus sylvestris*, *Quercus*, and *Salix*. Whilst Poaceae pollen remains relatively stable (53.61 % mean), Cyperaceae decreases (11.10 % mean). Herbaceous pollen remains generally low (5.43 % mean), though there is a small increase in *Plantago lanceolata* (1.40 % mean). Coprophilous fungal spore influx (76.06 spores cm^-2^ yr^-1^ mean) increases substantially in the uppermost samples (2 to 0 cm, *c.* -70 to -71 cal BP / 2020 to 2021 CE), peaking at 325.82 spores cm^-2^ yr^-1^, and microcharcoal (>50 µm) influx (1027.58 shards cm^-2^ yr^-1^ mean) follows a similar pattern, peaking at 3040.99 shards cm^-2^ yr^-1^.

Larkbarrow

The Larkbarrow pollen record (Figure S8) shows that whilst the site has remained open moorland for the last ~600 years, the character of the moorland vegetation has changed substantially during this time. Before the eighteenth century, the vegetation appears to have been characterised by a mixture of Poaceae, *Calluna vulgaris* and Cyperaceae, but during the eighteenth and nineteenth centuries, *C. vulgaris* appears to have been monodominant. From the early twentieth century, *C. vulgaris* declined, and Poaceae became monodominant. This latter change appears to be associated with increased evidence of burning (microcharcoal >50 µm). Tree and shrub pollen (*e.g.* *Quercus*, *Corylus avellana*-type, *Betula*) is present throughout, likely drawn from the surrounding landscape (>100 m).


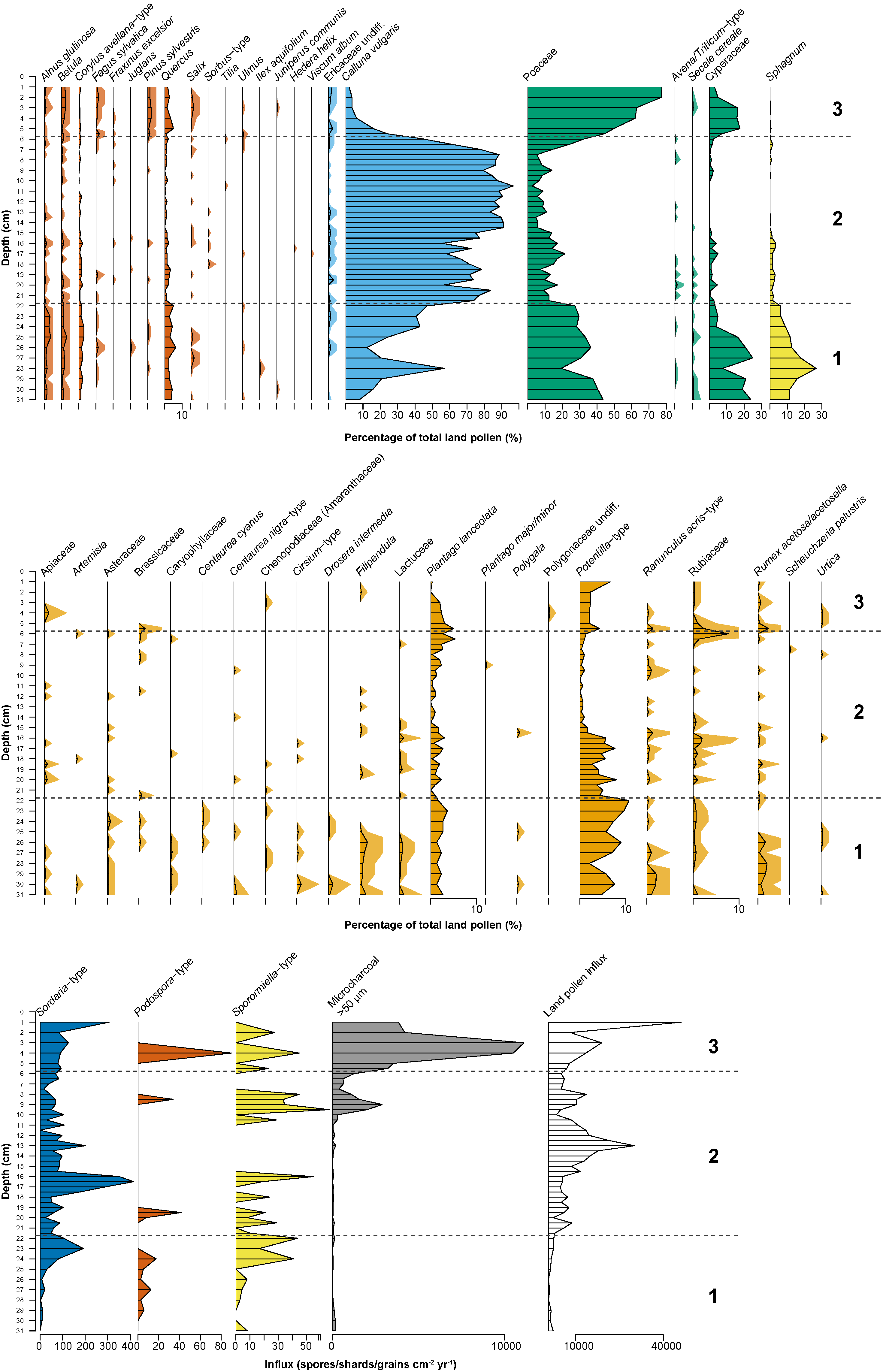


Figure S8 Summary of Larkbarrow pollen record. Numbers in bold refer to pollen zones objectively determined by CONISS (Grimm, 1987; Juggins, 2020)

Larkbarrow Zone 1 (31 to 21.5 cm, *c.* 551 to 263 cal BP / 1399 to 1686 CE) is characterised by high, but gradually declining, monocot relative abundances (47.77 % mean): mostly Poaceae (32.66 % mean), with some Cyperaceae (14.61 % mean). Tree and shrub pollen (9.66 % mean) is a mixture of *Quercus*, *Alnus glutinosa*, *Corylus avellana*-type and others. Relative abundances of *Calluna vulgaris* are moderate in this zone (28.90 % mean), and gradually increasing. Herbaceous taxa (13.31 % mean) are dominated by *Potentilla*-type, with some *Plantago lanceolata*. Relative abundances of *Sphagnum* are moderate here, and generally decreasing (12.83 % mean). Coprophilous fungal spore influx, represented by *Sordia*-type, *Podospora*-type and *Sporormiella*-type, is relatively low in this zone (63.37 spores cm^-2^ yr^-1^ mean), though increases from 24 cm (*c.* 334 cal BP / 1616 CE). Microcharcoal (>50 µm) influx is low in this zone (79.53 shards cm^-2^ yr^-1^ mean).

Larkbarrow Zone 2 (21.5 to 5.5 cm, *c.* 263 to -12.5 cal BP / 1686 to 1963 CE) is marked by a substantial increase in *Calluna vulgaris* relative abundances (77.88 % mean), and a substantial decrease in monocots (13.24 % mean), as well as decreases in trees and shrubs (3.17 % mean), herbaceous taxa (5.24 % mean) and *Sphagnum* (0.98 % mean). From 14.5 cm (*c.* 152 cal BP / 1758 CE), the monodominance of *C. vulgaris* further increased, with a peak of 96.70 % at 10.5 cm (*c.* 102 cal BP / 1848 CE), and all other pollen types decrease correspondingly. Cyperaceae decreases to the point of absence from 13.5 to 11 cm (*c.* 142 to 113 cal BP / 1808 to 1837 CE), and *Sphagnum* is absent or <2 % from 15 cm (*c.* 157 / 1793 CE) onwards. *C. vulgaris* begins to decline and monocots begin to increase after 7.5 cm (*c.* 32 cal BP / 1918 CE). Interestingly, *Scheuchzeria palustris* (‘Rannoch-rush’) briefly appears in the sequence at this depth. Coprophilous fungal spore influx is generally higher than the previous zone (113.55 spores cm^-2^ yr^-1^ mean) and it increases substantially from 17.5 to 15.5 cm (*c.* 186 to 162 cal BP / 1763 to 1787 CE), peaking at 16.5 cm (433.88 spores cm^-2^ yr^-1^, *c.* 178 cal BP / 1776 CE). Microcharcoal (>50 µm) influx remains low through most of this zone (385.62 shards cm^-2^ yr^-1^ mean), though there is a brief peak of 2891.54 shards cm^-2^ yr^-1^ at 9 cm (*c.* 68 cal BP / 1882 CE).

Larkbarrow Zone 3 (5.5 to 0 cm, *c.* -12.5 to -71 cal BP / 1963 to 2021 CE) is characterised by substantially lower *Calluna vulgaris* relative abundance (9.30 % mean) than the previous zone, and much greater monocot abundance (73.27 % mean), particularly Poaceae (62.25 % mean). Tree and shrub pollen is slightly more prominent (8.63 % mean), represented by a range of taxa (*e.g.* *Quercus*, *Betula*, *Fagus sylvatica*, *Pinus sylvestris*), as is herbaceous pollen (7.63 % mean), largely represented by *Potentilla*-type and *Plantago lanceolata*. Coprophilous fungal spore influx (160.68 spores cm^-2^ yr^-1^ mean) is generally higher than in the previous zone, and microcharcoal (>50 µm) influx is substantially greater (6079.79 shards cm^-2^ yr^-1^ mean), with a peak of 11138.55 shards cm^-2^ yr^-1^ at 3 cm (*c.* -49.7 cal BP / 2000 CE).

Little Ashcombe

The Little Ashcombe pollen record (Figure S9) shows that the site has remained open, grassy moorland for the last ~500 years, with subtle changes in the character of vegetation. Prior to the sixteenth century, the site appears to have been slightly wetter, with greater Cyperaceae and *Sphagnum* relative abundances. From the eighteenth century, *Sphagnum* declines and burning appears to increase in frequency. During the nineteenth century, *Sphagnum* becomes almost absent and there is a subtle change in herbaceous assemblages with less *Plantago lanceolata* and increasing *Potentilla*-type. *Rumex acetosa/acetosella* becomes more prominent during the twentieth century, alongside increased coprophilous fungal spore influx. *Sphagnum* spores also become absent more frequently during this period. Low quantities (<10 %) of tree and shrub pollen (*e.g.* *Quercus*, *Corylus avellana*-type) are present throughout, likely drawn from the surrounding landscape (>100 m).


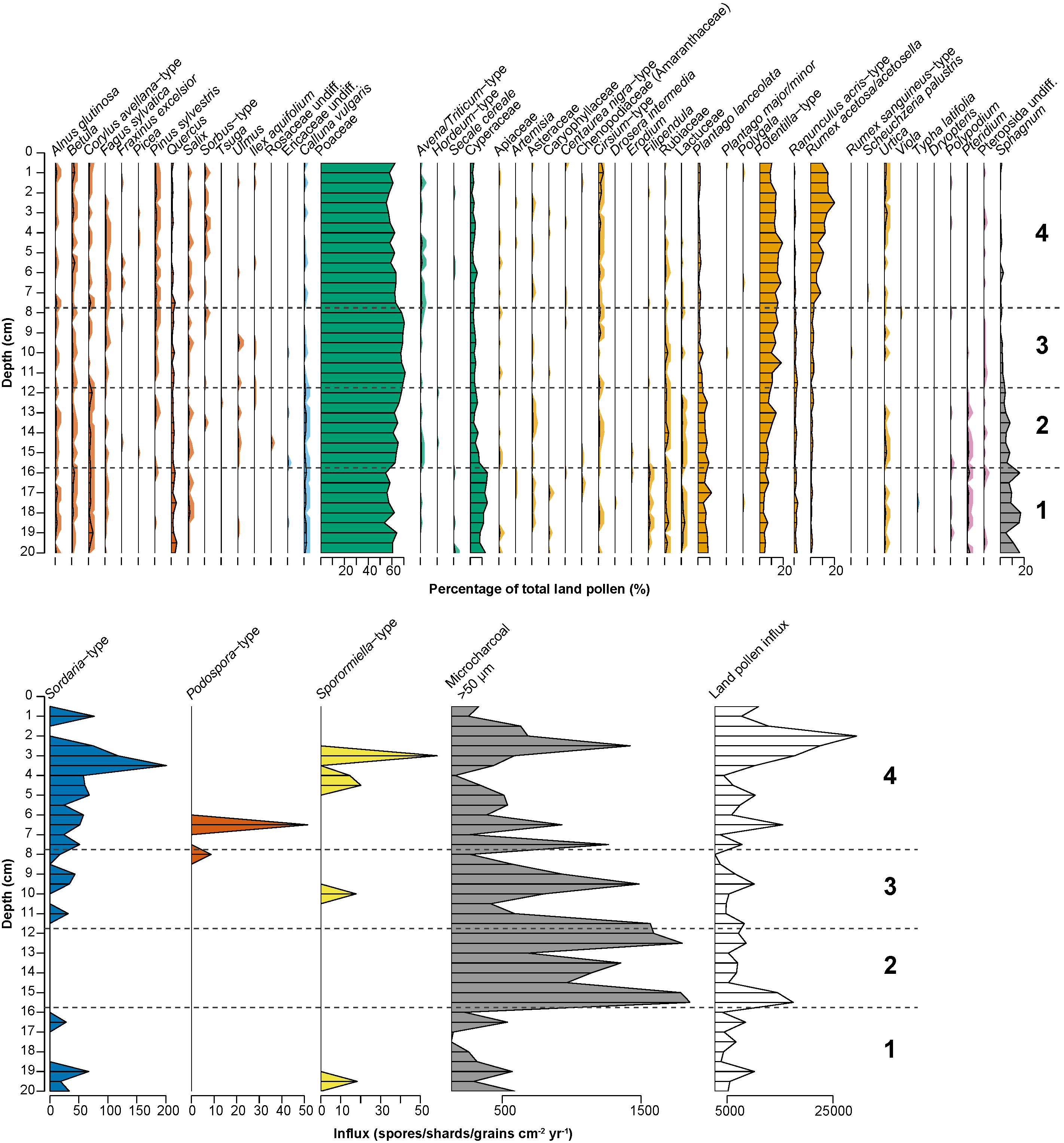


Figure S9 Summary of Little Ashcombe pollen record. Numbers in bold refer to pollen zones objectively determined by CONISS (Grimm, 1987; Juggins, 2020)

Little Ashcombe Zone 1 (20 to 15.5 cm, *c.* 428 to 250 cal BP / 1522 to 1700 CE) has the highest relative abundances of Cyperaceae (11.80 % mean) and *Sphagnum* (11.90 % mean) in the sequence. Poaceae values are at their lowest in this zone (58.51 % mean), though they exhibit little change through the sequence. Herbaceous taxa (20.53 % mean) include *Plantago lanceolata* and *Potentilla*-type, with *Filipendula*, *Galium*, Lactuceae and *Urtica* also present. Ericales (*Calluna vulgaris*, Ericaceae undiff.) are present at low levels (1.39 % mean), as are a range of tree and shrub taxa (7.48 % mean), including *Quercus* and *Corylus avellane*-type. Coprophilous fungal spore influx (18.41 spores cm^-2^ yr^-1^ mean), represented by *Sordia*- and *Sporormiella*-types, and microcharcoal (>50 µm) influx (341.00 shards cm^-2^ yr^-1^ mean) are both relatively low in this zone.

Little Ashcombe Zone 2 (15.5 to 11.5 cm, *c.* 250 to 120 cal BP / 1700 to 1830 CE) is similar to Zone 1 in terms herbaceous taxa (22.81 % mean), ericales (1.20 % mean) and trees and shrubs (6.06 % mean). Poaceae relative abundance is slightly higher (64.13 % mean), and both Cyperaceae (5.39 % mean) and *Sphagnum* (5.44 % mean) are lower than in Zone 1. Evidence of burning (microcharcoal >50 µm) increases substantially relative to the previous zone (1395.52 shards cm^-2^ yr^-1^ mean), whilst coprophilous fungal spores are absent.

Little Ashcombe Zone 3 (11.5 to 7.5 cm, *c.* 120 to 3 cal BP / 1830 to 1947 CE) is similar to previous zones in terms herbaceous taxa (21.63 % mean), ericales (0.33 % mean) and trees and shrubs (5.07 % mean). Poaceae (69.09 % mean) and Cyperaceae (3.67 % mean) remain similar to Zone 2, whilst *Sphagnum* shows further decline (1.53 % mean), and is briefly absent (8.5 cm, *c.* 27 cal BP / 1923 CE). Amongst herbaceous taxa, *Potentilla­*-type (12.97 % mean) increases slightly in this zone, whilst *Plantago lanceolata* decreases (2.40 % mean). Coprophilous fungal spores are present at low levels in this zone (19.11 spores cm^-2^ yr^-1^ mean), represented by *Sordaria*-type, *Podospora*-type and *Sporormiella*-type. Evidence of burning (microcharcoal >50 µm) shows general decrease relative to the previous zone (829.06 shards cm^-2^ yr^-1^ mean), but similar peaks remain (*e.g.* 1486.04 shards cm^-2^ yr^-1^ at 9.5 cm, *c.* 56 cal BP / 1894 CE).

In Little Ashcombe Zone 4 (7.5 to 0 cm, *c.* 3 to -71 cal BP / 1947 to 2021 CE) Poaceae relative abundances are lower than in the previous zone (60.23 % mean), whilst relative abundances of herbaceous taxa are higher (30.09 % mean). This is driven by a substantial increase in *Rumex acetosa/acetosella* (10.59 % mean), alongside small increases in *Cirsium*-type (1.41 % mean) and *Urtica* (0.88 % mean). *Scheuchzeria palustris* (‘Rannoch-rush’) briefly appears in this zone (7 cm, *c.* -7 cal BP / 1957 CE). *Sphagnum* is either absent or nearly absent (<1 %) for much of this zone (0.60 % mean), and ericales show a similar pattern (0.29 % mean). Coprophilous fungal spore influx is notably higher and more consistent than in previous zones (67.44 spores cm^-2^ yr^-1^ mean), primarily represented by *Sordaria*-type, with *Podospora*- and *Sporormiella*-types occasionally present. Evidence of burning (microcharcoal >50 µm) shows further general decrease relative to previous zones (582.79 shards cm^-2^ yr^-1^ mean), though similar peaks continue to occur (*e.g.* 1421.47 shards cm^-2^ yr^-1^ at 2.5 cm, *c.* -54 cal BP / 2004 CE).

The Chains

The pollen data for The Chains (Figure S10) demonstrates that the site has been open moorland for at least the last ~600 years, with a high proportion of monocots (Poaceae and Cyperaceae), and some heathers (*Calluna vulgaris*, Ericaceae undiff.) and herbaceous taxa (*Plantago lanceolata*, with Apiaceae, *Potentilla*-type, *Rumex acetosa/acetosella* and others). Tree and shrub pollen (*e.g.* *Corylus avellana*-type, *Quercus*) is also present, likely drawn from the surrounding landscape (>100 m). During the early nineteenth century, the character of moorland on The Chains appears to have changed towards a higher proportion of monocots and lower proportion of *C. vulgaris*, alongside marginally increased diversity amongst heather taxa.


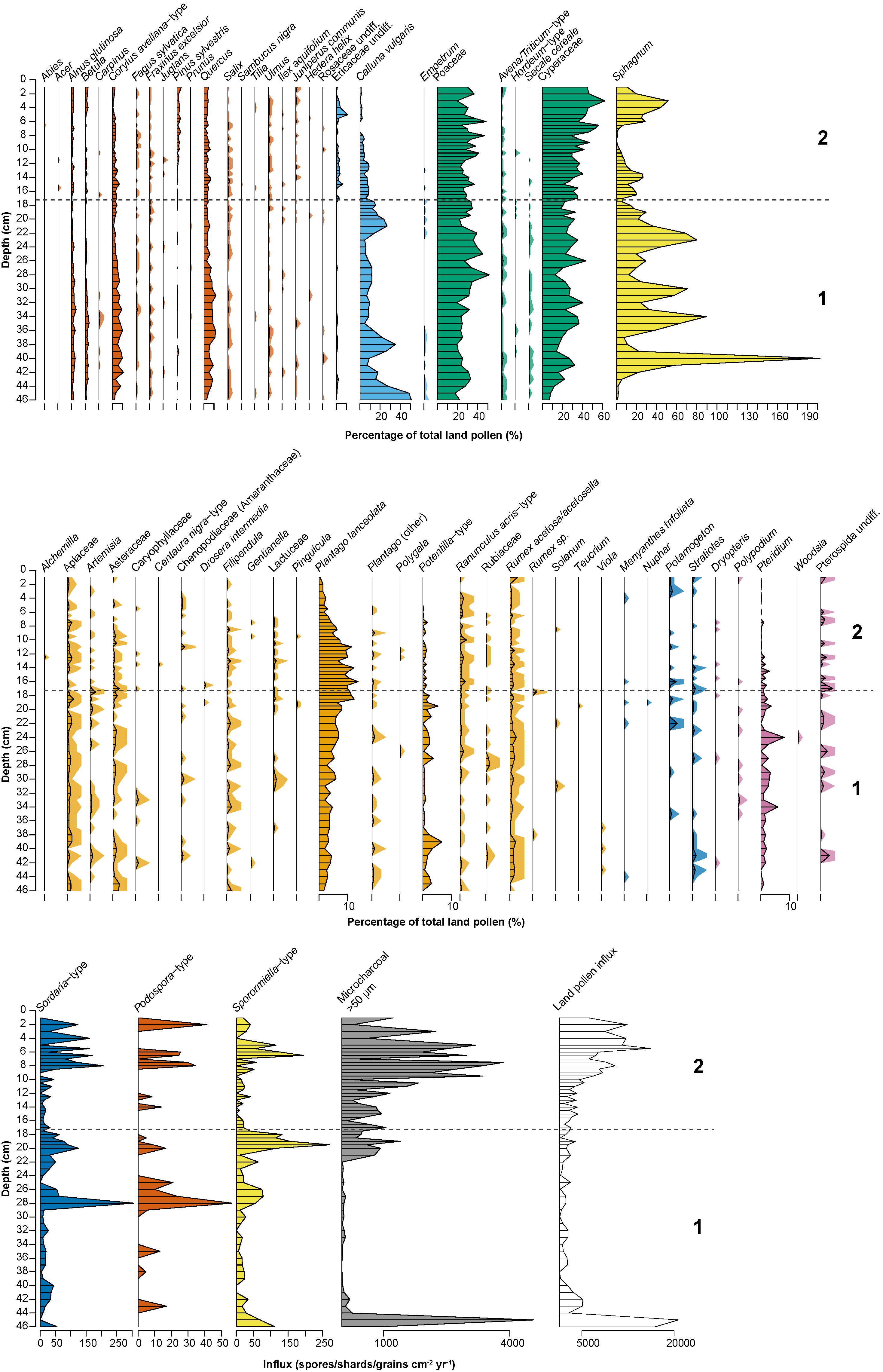


Figure S10 Summary of The Chains pollen record. Numbers in bold refer to pollen zones objectively determined by CONISS (Grimm, 1987; Juggins, 2020)

The Chains Zone 1 (46 to 17 cm, *c.* 545 to 148 cal BP / 1405 to 1802 CE) is characterised by fluctuating *Calluna vulgaris* (min: 4.65 %, max: 50.99 %, mean: 16.288 %) and *Sphagnum* (min: 1.33 %, max: 202.33 %, mean: 36.13 %). Higher values of *C. vulgaris* in this zone generally occur when *Sphagnum* values are lower. Cyperaceae (min: 6.95 %, max: 43.38 %, mean: 24.05 %) also fluctuates, with higher values broadly corresponding to higher *Sphagnum* values. Collectively, these suggest fluctuating wetness. Poaceae (min: 17.33 %, max: 51.33 %, mean: 29.42 %) shows a general increase in this zone, whilst tree and shrub pollen (min: 8.94 %, max: 30.59 %, 17.46 % mean), declines. Trees and shrubs are primarily represented by *Corylus avellana*-type and *Quercus*, with *Alnus glutinosa*, *Betula* and a range of other taxa also present. Herbaceous pollen (11.15 % mean) is relatively stable, and is comprised of Apiaceae, *Plantago lanceolata*, *Potentilla*-type, *Rumex acestosa/acetosella* and other taxa. Evidence of burning (microcharcoal >50 µm) is limited during this zone (375.34 shards cm^-2^ yr^-1^ mean), asides from a brief spike (4556.83 shards cm^-2^ yr^-1^) at 45 cm (*c.* 540 cal BP / 1410 CE), though it increases towards the end of the zone. Coprophilous fungal spore influx generally remains low (96.25 spores cm^-2^ yr^-1^ mean), with two notable spikes at 28 cm (418.86 spores cm^-2^ yr^-1^: *c.* 307 cal BP / 1643 CE) and 19.5 cm (371.07 spores cm^-2^ yr^-1^: *c.* 178 cal BP / 1772 CE).

In The Chains Zone 2 (17 to 0 cm, *c.* 148 to Y cal BP / 1802 to 2017 CE), monocots generally increase (69.70 % mean), primarily as a result of increasing Cyperaceae (38.30 % mean). Heathers decrease in abundance overall (6.28 % mean), as a result of declining *Calluna vulgaris* (3.97 % mean). However, whilst *C. vulgaris* was monodominant amongst heathers in the previous zone, other heathers (Ericaceae undiff.) are also present in this zone (1.67 % mean). Tree and shrub abundance remains relatively stable (13.33 % mean), characterised by similar taxa to the previous zone, although with increasing *Pinus sylverstris*. Herbaceous taxa (10.71 % mean) decline in this zone, primarily representing a decline in *Plantago lanceolata*. Evidence of burning (microcharcoal >50 µm) (1457.26 shards cm^-2^ yr^-1^ mean) in the first part of this zone largely continues from moderate increase at the end of the previous zone, before increasing substantially at 9.5 cm (*c.* 58 cal BP / 1892 CE) and peaking at 7.5 cm (*c.* 33 cal BP / 1917 CE) (3848.5 shards cm^-2^ yr^-1^). Coprophilous fungal spore influx is similar to the previous zone (87.19 spores cm^-2^ yr^-1^ mean), but peaks around a similar point to microcharcoal: 390.55 spores cm^-2^ yr^-1^ at 6.5 cm (*c.* 21 cal BP / 1929 CE).

*Cluster analysis*

Clusters have been discussed in terms of their vegetation composition and relative timings in the main body of text. In Figure S11 we show that whilst Clusters 1 and 5 are primarily (but not exclusively) composed of sub-samples from single sites (Little Ashcombe and Larkbarrow, respectively), Clusters 2, 3 and 4 are substantially more mixed in this context. This indicates that whilst individual site characteristics (*e.g.* hydrology) are a notable control on vegetation, other controls (*e.g.* disturbance regimes) are also important.


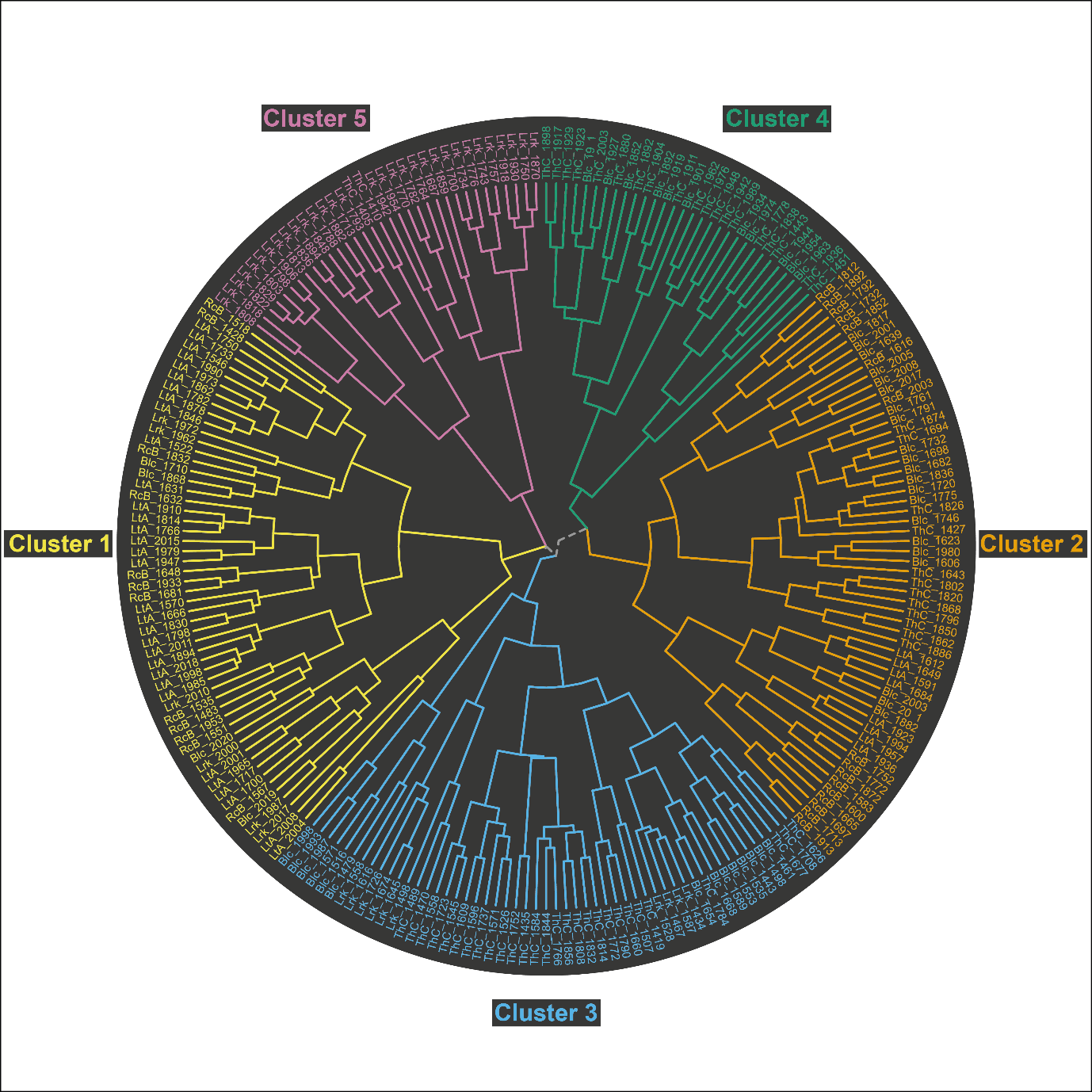


Figure S11 Graphical summary of clusters. Blc: Blackpitts; Lrk: Larkbarrow; LtA: Little Ashcombe; RcB: Ricksy Ball; ThC: The Chains. Numbers refer to modelled dates per sub-sample (CE).

*Regression analyses*

The following tables (Tables S6 to S8) provide summaries of generalised additive models (GAMs) used in addressing the primary research questions (also summarised graphically in Figures 7 to 9).

Table S6 Summary of a generalised additive model (GAM) (Figure 7) showing the estimated effects of several explanatory variables on Sphagnum influx rates (spores cm^-2^ yr^-1^) (n=208)

| Parametric coefficients |  |  |  | |  | |  | |
| --- | --- | --- | --- | --- | --- | --- | --- | --- |
| **Covariate** | **Category** | **Estimate** | **Standard Error** | | **P-value** | | **Significance** | |
| Site type  *(Ombrotrophic)* | Soligeneous | -255.216 | 91.443 | | 0.005 | | ** | |
| Drainage  *(Pre-drainage)* | Post-drainage | -171.240 | 45.426 | | 0.000 | | *** | |
|  |  |  |  | |  | |  | |
| Approximate significance of smooth and random effect terms | | | |  | |  | |  |
| **Covariate** | **Effect** | **EDF** | **P-value** | | **Significance** | | | |
| Summer precipitation | Smooth | 0.310 | 0.211 | |  | |  | |
| Coprophilous fungal spores | Smooth | 0.000 | 0.509 | |  | |  | |
| Microcharcoal | Smooth | 0.000 | 0.461 | |  | |  | |
| Site | Random | 2.244 | 0.009 | | ** | |  | |
| Significance codes: ‘***’p < 0.001; ‘**’ p < 0.01; ‘*’ p < 0.05; ‘-’ p < 0.1; ‘ ’ p > 0.1 | | | | | | | |  |

Table S7 Summary of a generalised additive model (GAM) (Figure 8) showing the estimated effects of several explanatory variables on graminoid monocot influx rates (pollen grains cm^-2^ yr^-1^) (n=208)

| Parametric coefficients |  |  |  | |  | |  | |
| --- | --- | --- | --- | --- | --- | --- | --- | --- |
| **Covariate** | **Category** | **Estimate** | **Standard Error** | | **P-value** | | **Significance** | |
| Site type  *(Ombrotrophic)* | Soligeneous | 538.654 | 1251.522 | | 0.667 | |  | |
| Drainage  *(Pre-drainage)* | Post-drainage | -79.791 | 173.308 | | 0.645 | |  | |
|  |  |  |  | |  | |  | |
| Approximate significance of smooth and random effect terms | | | |  | |  | |  |
| **Covariate** | **Effect** | **EDF** | **P-value** | | **Significance** | | | |
| Summer precipitation | Smooth | 0.000 | 0.426 | |  | |  | |
| Coprophilous fungal spores | Smooth | 0.901 | 0.004 | | ** | |  | |
| Microcharcoal | Smooth | 1.234 | 0.000 | | *** | |  | |
| Site | Random | 2.949 | 0.000 | | *** | |  | |
| Significance codes: ‘***’p < 0.001; ‘**’ p < 0.01; ‘*’ p < 0.05; ‘-’ p < 0.1; ‘ ’ p > 0.1 | | | | | | | |  |

Table S8 Summary of a generalised additive model (GAM) (Figure 9) showing the estimated effects of several explanatory variables on non-arboreal taxon richness (n=208)

| Parametric coefficients |  |  |  | |  | |  | |
| --- | --- | --- | --- | --- | --- | --- | --- | --- |
| **Covariate** | **Category** | **Estimate** | **Standard Error** | | **P-value** | | **Significance** | |
| Site type  *(Ombrotrophic)* | Soligeneous | -1.395 | 1.619 | | 0.390 | |  | |
| Drainage  *(Pre-drainage)* | Post-drainage | -2.270 | 0.432 | | 0.000 | | *** | |
|  |  |  |  | |  | |  | |
| Approximate significance of smooth and random effect terms | | | |  | |  | |  |
| **Covariate** | **Effect** | **EDF** | **P-value** | | **Significance** | | | |
| Summer precipitation | Smooth | 0.000 | 0.455 | |  | |  | |
| Coprophilous fungal spores | Smooth | 2.253 | 0.000 | | *** | |  | |
| Microcharcoal | Smooth | 0.000 | 0.975 | |  | |  | |
| Site | Random | 2.770 | 0.000 | | *** | |  | |
| Significance codes: ‘***’p < 0.001; ‘**’ p < 0.01; ‘*’ p < 0.05; ‘-’ p < 0.1; ‘ ’ p > 0.1 | | | | | | | |  |

The following tables (Tables S9 and S10) provide tabular summaries of generalised additive models (GAMs) used in sensitivity testing.

Table S9 Summary of a generalised additive model (GAM) showing the estimated effects of several explanatory variables on Sphagnum influx rates (spores cm^-2^ yr^-1^) during the period 1750 to 1900 CE (n=72)

| Parametric coefficients |  |  |  | |  | |  | |
| --- | --- | --- | --- | --- | --- | --- | --- | --- |
| **Covariate** | **Category** | **Estimate** | **Standard Error** | | **P-value** | | **Significance** | |
| Site type  *(Ombrotrophic)* | Soligeneous | -318.752 | 93.624 | | 0.001 | | *** | |
| Drainage  *(Pre-drainage)* | Post-drainage | -125.631 | 47.010 | | 0.008 | | ** | |
|  |  |  |  | |  | |  | |
| Approximate significance of smooth and random effect terms | | | |  | |  | |  |
| **Covariate** | **Effect** | **EDF** | **P-value** | | **Significance** | | | |
| Summer precipitation | Smooth | 0.000 | 0.901 | |  | |  | |
| Coprophilous fungal spores | Smooth | 0.000 | 0.453 | |  | |  | |
| Microcharcoal | Smooth | 0.000 | 0.471 | |  | |  | |
| Site | Random | 2.137 | 0.016 | | * | |  | |
| Significance codes: ‘***’p < 0.001; ‘**’ p < 0.01; ‘*’ p < 0.05; ‘-’ p < 0.1; ‘ ’ p > 0.1 | | | | | | | |  |

Table S10 Summary of a generalised additive model (GAM) showing the estimated effects of several explanatory variables on non-arboreal taxon richness (n=208), with total coprophilous fungal spores replaced with only Sordaria-type spores

| Parametric coefficients |  |  |  | |  | |  | |
| --- | --- | --- | --- | --- | --- | --- | --- | --- |
| **Covariate** | **Category** | **Estimate** | **Standard Error** | | **P-value** | | **Significance** | |
| Site type  *(Ombrotrophic)* | Soligeneous | -1.167 | 1.481 | | 0.432 | |  | |
| Drainage  *(Pre-drainage)* | Post-drainage | -2.250 | 0.436 | | 0.000 | | *** | |
|  |  |  |  | |  | |  | |
| Approximate significance of smooth and random effect terms | | | |  | |  | |  |
| **Covariate** | **Effect** | **EDF** | **P-value** | | **Significance** | | | |
| Summer precipitation | Smooth | 0.001 | 0.445 | |  | |  | |
| *Sordaria*-type spores | Smooth | 2.148 | 0.000 | | *** | |  | |
| Microcharcoal | Smooth | 0.000 | 0.743 | |  | |  | |
| Site | Random | 2.723 | 0.000 | | *** | |  | |
| Significance codes: ‘***’p < 0.001; ‘**’ p < 0.01; ‘*’ p < 0.05; ‘-’ p < 0.1; ‘ ’ p > 0.1 | | | | | | | |  |

***References for Appendix: Supporting information***

Appleby, P. G. (2001). Chronostratigraphic Techniques in Recent Sediments. In W. M. Last & J. P. Smol (Eds.), *Tracking Environmental Change Using Lake Sediments. Volume 1: Basin Analysis, Coring, and Chronological Techniques.* (pp. 171–203). Dordrecht, The Netherlands: Kluwer Academic Publishers.

Appleby, P. G., & Oldfield, F. (1978). The calculation of lead-210 dates assuming a constant rate of supply of unsupported 210Pb to the sediment. *Catena*, *5*(1), 1–8. doi: 10.1016/S0341-8162(78)80002-2

Aquino-López, M. A., Blaauw, M., Christen, J. A., & Sanderson, N. K. (2018). Bayesian Analysis of ^210^Pb Dating. *Journal of Agricultural, Biological, and Environmental Statistics*, *23*(3), 317–333. doi: 10.1007/s13253-018-0328-7

Baker, A. G., Bhagwat, S. A., & Willis, K. J. (2013). Do dung fungal spores make a good proxy for past distribution of large herbivores? *Quaternary Science Reviews*, *62*, 21–31. doi: 10.1016/j.quascirev.2012.11.018

Bennett, K. D. (1994). *Annotated catalogue of pollen and pteridophyte spore types (unpublished)*. Retrieved from https://www.researchgate.net/profile/Keith-Bennett-2/publication/245416986_Annotated_Catalogue_of_Pollen_and_Pteridophyte_Spore_Types_of_the_British_Isles/links/5481602b0cf22525dcb60b7c/Annotated-Catalogue-of-Pollen-and-Pteridophyte-Spore-Types-of-the-Bri

Blaauw, M., Christen, J. A., & Aquino-López, M. A. (2021). *rplum: Bayesian Age-Depth Modelling of Cores Dated by Pb-210.* Retrieved from https://cran.r-project.org/package=rplum

Blaauw, M., Christen, J. A., & Aquino-López, M. A. (2022). *rbacon: Age-Depth Modelling using Bayesian Statistics*. Retrieved from https://cran.r-project.org/package=rbacon

Blockley, S. P. E., Pyne-O’Donnell, S. D. F., Lowe, J. J., Matthews, I. P., Stone, A., Pollard, A. M., … Molyneux, E. G. (2005). A new and less destructive laboratory procedure for the physical separation of distal glass tephra shards from sediments. *Quaternary Science Reviews*, *24*(16–17), 1952–1960. doi: 10.1016/j.quascirev.2004.12.008

Bray, J. R., & Curtis, J. T. (1957). An Ordination of the Upland Forest Communities of Southern Wisconsin. *Ecological Monographs*, *27*(4), 325–349. doi: 10.2307/1942268

Chambers, F. M., Daniell, J. R. G., Hunt, J. B., Molloy, K., & O’Connell, M. (2004). Tephrostratigraphy of An Loch Mór, Inis Oírr, western Ireland: Implications for Holocene tephrochronology in the northeastern Atlantic region. *Holocene*, *14*(5), 703–720. doi: 10.1191/0959683604hl749rp

Charman, D. J. (2007). Summer water deficit variability controls on peatland water-table changes: Implications for Holocene palaeoclimate reconstructions. *The Holocene*, *17*(2), 217–227. doi: 10.1177/0959683607075836

Djamali, M., & Cilleros, K. (2020). Statistically significant minimum pollen count in Quaternary pollen analysis; the case of pollen-rich lake sediments. *Review of Palaeobotany and Palynology*, *275*, 104156. doi: 10.1016/j.revpalbo.2019.104156

Eden, D., Froggatt, P., & McIntosh, P. (1992). The distribution and composition of volcanic glass in late Quaternary loess deposits of southern South Island, New Zealand, and some possible correlations. *New Zealand Journal of Geology and Geophysics*, *35*(1), 69–79.

Fyfe, R. M., Woodbridge, J., & Roberts, C. N. (2018). Trajectories of change in Mediterranean Holocene vegetation through classification of pollen data. *Vegetation History and Archaeobotany*, *27*(2), 351–364. doi: 10.1007/s00334-017-0657-4

Grimm, E. C. (1987). CONISS: a FORTRAN 77 program for stratigraphically constrained cluster analysis by the method of incremental sum of squares. *Computers and Geosciences*, *13*(1), 13–35. doi: 10.1016/0098-3004(87)90022-7

Hayward, C. (2012). High spatial resolution electron probe microanalysis of tephras and melt inclusions without beam-induced chemical modification. *The Holocene*, *22*(1), 119–125.

Holmes, J. A., Leuenberger, M., Molloy, K., & O’Connell, M. (2020). Younger Dryas and Holocene environmental change at the Atlantic fringe of Europe derived from lake-sediment stable-isotope records from western Ireland. *Boreas*, *49*(2), 233–247. doi: 10.1111/bor.12425

Hunt, J. B., & Hill, P. G. (1993). Tephra geochemistry: a discussion of some persistent analytical problems. *The Holocene*, *3*(3), 271–278.

Juggins, S. (2020). *rioja: Analysis of Quaternary Science Data*. Retrieved from https://cran.r-project.org/package=rioja

Loader, N. J., Young, G. H. F., McCarroll, D., Davies, D., Miles, D., & Bronk Ramsey, C. (2020). Summer precipitation for the England and Wales region, 1201–2000 ce, from stable oxygen isotopes in oak tree rings. *Journal of Quaternary Science*, *35*(6), 731–736. doi: 10.1002/jqs.3226

Mooney, S. D., & Tinner, W. (2011). The analysis of charcoal in peat and organic sediments. *Mires and Peat*, *7*(1941), 1–18. Retrieved from http://pixelrauschen.de/wbmp/media/map07/map_07_09.pdf

Moore, P. D., Webb, J., & Collinson, M. E. (1991). *Pollen Analysis*. Blackwell, Oxford.

Mottl, O., Grytnes, J. A., Seddon, A. W. R., Steinbauer, M. J., Bhatta, K. P., Felde, V. A., … Birks, H. J. B. (2021). Rate-of-change analysis in paleoecology revisited: A new approach. *Review of Palaeobotany and Palynology*, *293*. doi: 10.1016/j.revpalbo.2021.104483

Oksanen, J., Simpson, G. L., Blanchet, F., Kindt, R., Legendre, P., Minchin, P., … Weedon, J. (2022). *vegan: Community Ecology Package. R package version 2.6-2*. Retrieved from https://cran.r-project.org/package=vegan

Ombashi, H. (2019). *A high resolution palaeoecological study of land use change during late prehistory on Exmoor*. University of Plymouth.

Orwin, C. S., & Sellick, R. J. (1970). *The Reclamation of Exmoor Forest* (Second Edi). Newton Abbot: David and Charles.

Perrotti, A. G., & van Asperen, E. N. (2018). Dung fungi as a proxy for megaherbivores: opportunities and limitations for archaeological applications. *Vegetation History and Archaeobotany*, *0*(0), 1–12. doi: 10.1007/s00334-018-0686-7

Piotrowska, N., Blaauw, M., Mauquoy, D., & Chambers, F. M. (2011). Constructing deposition chronologies for peat deposits using radiocarbon dating. *Mires and Peat*, *7*(10), 1–14. doi: 10.1111/j.1365-2486.2009.01920.x

Plunkett, G., & Pilcher, J. R. (2018). Defining the potential source region of volcanic ash in northwest Europe during the Mid- to Late Holocene. *Earth-Science Reviews*, *179*(January), 20–37. doi: 10.1016/j.earscirev.2018.02.006

R Core Team. (2022). *R: A language and environment for statistical computing*. R Foundation for Statistical Computing. Retrieved from https://www.r-project.org/

Reimer, P. J., Austin, W. E. N., Bard, E., Bayliss, A., Blackwell, P. G., Bronk Ramsey, C., … Talamo, S. (2020). The IntCal20 Northern Hemisphere Radiocarbon Age Calibration Curve (0-55 cal kBP). *Radiocarbon*, *62*(4), 725–757. doi: 10.1017/RDC.2020.41

Rowney, F. M., Fyfe, R. M., Anderson, P., Barnett, R., Blake, W., Daley, T., … Smith, D. (2022). Ecological consequences of historic moorland ‘improvement.’ *Biodiversity and Conservation*. doi: 10.1007/s10531-022-02479-6

Stockmarr, J. (1971). Tablets with spores used in absolute pollen analysis. *Pollen et Spores*, *XIII*(January 1971), 615–621.

Turney, C. S. M. (1998). Extraction of rhyolitic component of Vedde microtephra from minerogenic lake sediments. *Journal of Paleolimnology*, *19*(2), 199–206.

Väliranta, M., Oinonen, M., Seppä, H., Korkonen, S., Juutinen, S., & Tuittila, E.-S. (2014). Unexpected Problems in AMS 14C Dating of Fen Peat. *Radiocarbon*, *56*(1), 95–108. doi: 10.2458/56.16917

van Asperen, E. N., Kirby, J. R., & Hunt, C. O. (2016). The effect of preparation methods on dung fungal spores: Implications for recognition of megafaunal populations. *Review of Palaeobotany and Palynology*, *229*, 1–8. doi: 10.1016/j.revpalbo.2016.02.004

Wilson-North, R. (2017). The rediscovery of the Knight family archive and its importance to Exmoor. *Somerset Archaeology and Natural History*, *161*, 189–194.

Wood, J. R., & Wilmshurst, J. M. (2013). Accumulation rates or percentages? How to quantify *Sporormiella* and other coprophilous fungal spores to detect late Quaternary megafaunal extinction events. *Quaternary Science Reviews*, *77*, 1–3. doi: 10.1016/j.quascirev.2013.06.025
